# The impact of P-gp functionality on non-steady state relationships between CSF and brain extracellular fluid

**DOI:** 10.1007/s10928-013-9314-4

**Published:** 2013-03-29

**Authors:** Joost Westerhout, Jean Smeets, Meindert Danhof, Elizabeth C. M. de Lange

**Affiliations:** 1 Department of Pharmacology, Leiden/Amsterdam Center for Drug Research, Einsteinweg 55, 2333 CC Leiden, The Netherlands; 2 LAP&P Consultants B.V., Leiden, The Netherlands

**Keywords:** Pharmacokinetics, Blood–brain barrier, P-glycoprotein, Systems-based pharmacokinetic modeling, Brain extracellular fluid, Cerebrospinal fluid

## Abstract

In the development of central nervous system (CNS)-targeted drugs, the prediction of human CNS target exposure is a big challenge. Cerebrospinal fluid (CSF) concentrations have often been suggested as a ‘good enough’ surrogate for brain extracellular fluid (brain_ECF_, brain target site) concentrations in humans. However, brain anatomy and physiology indicates prudence. We have applied a multiple microdialysis probe approach in rats, for continuous measurement and direct comparison of quinidine kinetics in brain_ECF_, CSF, and plasma. The data obtained indicated important differences between brain_ECF_ and CSF kinetics, with brain_ECF_ kinetics being most sensitive to P-gp inhibition. To describe the data we developed a systems-based pharmacokinetic model. Our findings indicated that: (1) brain_ECF_- and CSF-to-unbound plasma AUC_0–360_ ratios were all over 100 %; (2) P-gp also restricts brain intracellular exposure; (3) a direct transport route of quinidine from plasma to brain cells exists; (4) P-gp-mediated efflux of quinidine at the blood–brain barrier seems to result of combined efflux enhancement and influx hindrance; (5) P-gp at the blood–CSF barrier either functions as an efflux transporter or is not functioning at all. It is concluded that in parallel obtained data on unbound brain_ECF_, CSF and plasma concentrations, under dynamic conditions, is a complex but most valid approach to reveal the mechanisms underlying the relationship between brain_ECF_ and CSF concentrations. This relationship is significantly influenced by activity of P-gp. Therefore, information on functionality of P-gp is required for the prediction of human brain target site concentrations of P-gp substrates on the basis of human CSF concentrations.

**Electronic supplementary material:**

The online version of this article (doi:10.1007/s10928-013-9314-4) contains supplementary material, which is available to authorized users.

## Introduction

To be able to predict desired or undesired central nervous system (CNS) drug effects in humans, a mechanistic understanding is needed of the individual contributions of the processes involved in brain target site distribution and ultimately drug effects. With the unbound drug concentrations at the brain target site being responsible for the (un)wanted effect it is important to be able to determine or predict unbound drug concentrations at their site of action.

During the preclinical phase of drug development several techniques can be applied to determine or predict brain target site concentrations, which are often closely linked, or equal, to brain extracellular fluid (brain_ECF_) concentrations [1, 2]. However, most of the preclinical techniques have very limited applicability in the extrapolation of preclinical findings to the human situation [3–5].

Cerebrospinal fluid (CSF) concentrations are often considered to be the best available surrogate for brain target site concentrations in humans [6–10]. It is often assumed that CSF concentrations readily equilibrate with brain ECF concentrations due to the lack of a physical barrier between these sites [11]. However, due to qualitative and quantitative differences in processes that govern the pharmacokinetics (PK) of drugs in the brain, a generally applicable relationship between CSF concentrations and brain ECF concentrations does not exist [5, 12–14].

Transport of drugs into and out of the brain is not solely governed by the blood–brain barriers [the blood–brain barrier (BBB) and the blood–CSF barrier (BCSFB)], but also by the anatomy of the brain and physiological processes. In combination with drug specific properties [15–19], this determines the concentrations of a drug within a specific part of the CNS, including the target site concentrations, which we are ultimately interested in.

We have previously shown that even for acetaminophen, a model compound for passive transport into, within and out of the brain, differences exist between CSF and brain_ECF_ kinetics [20]. For compounds subjected to active transport at the level of the brain barriers, such as by P-gp, differences between brain_ECF_ and CSF are anticipated to be larger. With P-gp localized at both the luminal and abluminal membranes of capillary endothelial cells, as well as to adjacent pericytes and astrocytes [21], this suggests that P-gp may regulate drug transport processes in the entire brain at both the cellular and subcellular level. In contrast, P-gp presence and localization at the BCSFB is still subject of debate, with the only report of presence at the apical surface of the choroid plexus epithelial cells in the rat by Rao and colleagues [22]. Furthermore, it has been well established that P-gp functions as an efflux transporter at the BBB [23–26], whereas, there has been some evidence that P-gp could function as an influx transporter at the BCSFB [22, 27]. This could result in significant differences between concentrations at the brain target site and in CSF for compounds that are substrates for P-gp mediated transport.

The presence of P-gp at multiple sites, with in part a yet uncertain transport direction, could have major implications for the predictability of human brain_ECF_ concentrations on the basis of human CSF concentrations for compounds that are substrates for P-gp. Consequently, to be able to predict human brain_ECF_ concentrations on the basis of human CSF concentrations, one should first understand the mechanisms that determine the relationship between CSF concentrations and brain_ECF_ concentrations.

Previous studies have indicated that, under steady-state conditions, CSF concentrations were comparable to steady-state brain_ECF_ concentrations for compounds that freely diffuse across the BBB and BCSFB, but may differ for compounds that are substrate for the various active transport systems at the BBB and BCSFB [6–10]. CSF and brain_ECF_ concentration ratios were considered comparable if smaller than threefold, and assumed to be of little pharmacological consequence.

However, we have previously questioned this arbitrary threefold range in ratio of CSF and brain_ECF_ (target site) kinetics, especially with regard to the unknown impact of the steady-state situation versus the more realistic multiple dosing conditions (troughs and peaks), the unknown of the changes therein in disease conditions as well as the unknown impact of this range on pharmacodynamics [5]. These unknowns need to be investigated before we can really predict human target site PK and finally CNS effects.

Using the multiple intracerebral probe microdialysis approach [striatum (ST), lateral ventricle (LV), and cisterna magna (CM)] with parallel blood sampling, continuous measurement and direct comparison of changes in concentrations in plasma, brain_ECF_ and CSF kinetics of quinidine, a well-known P-gp substrate [28–32], could be assessed, following a short infusion of 10 and 20 mg/kg, with and without co-administration of the P-gp blocker tariquidar [33, 34]. Mathematical modelling was applied to the data to result in a number of key findings.

## Materials and methods

### Chemicals

Tariquidar (XR9576, TQD) was obtained from Xenova Group PLC (Cambridge, England) or API Services Inc. (Westford, USA). Quinidine, quinidine sulfate dehydrate, quinidine hemi sulfate and quinine hemi sulfate were obtained from Sigma Aldrich (Zwijndrecht, The Netherlands). Triethyl amine was obtained from J.T. Baker (Deventer, The Netherlands). Boric acid and orthophosphoric acid 85 % were obtained from Merck (Darmstadt, Germany). Methyl tert-butyl ether was obtained from Biosolve B.V. (Valkenswaard, The Netherlands). Isoflurane was obtained from Pharmachemie B.V. (Haarlem, The Netherlands). Saline and 5 % glucose were obtained from the Leiden University Medical Centre pharmacy (Leiden, The Netherlands). Microdialysis perfusion fluid was prepared as previously described [20], containing 140.3 mM sodium, 2.7 mM potassium, 1.2 mM calcium, 1.0 mM magnesium and 147.7 mM chloride.

### Animals

The study protocol was approved by the Animal Ethics Committee of Leiden University (UDEC nr. 07142) and all animal procedures were performed in accordance with Dutch laws on animal experimentation. A total of 60 male Wistar WU rats (225–275 g, Charles River, Maastricht, The Netherlands) were randomly divided into two groups; the first group (*n* = 12) was used for the determination of the in vivo microdialysis probe recovery; the second group (*n* = 48) was used for brain disposition experiments. This second group was further divided into four subgroups, designated for 10 or 20 mg/kg quinidine with or without co-administration of tariquidar (10^−^, 10^+^, 20^−^ and 20^+^).

After arrival, all animals were housed in groups for 5–7 days (Animal Facilities, Gorlaeus Laboratories, Leiden, The Netherlands), under standard environmental conditions (ambient temperature 21 °C; humidity 60 %; 12/12 h light/dark cycle, background noise, daily handling), with ad libitum access to food (Laboratory chow, Hope Farms, Woerden, The Netherlands) and acidified water. Between surgery and experiments, the animals were kept individually in Makrolon type three cages for 7 days to recover from the surgical procedures.

### Surgery

All surgical procedures were performed as described by Westerhout et al. [20]. In short, cannulas were implanted in the left femoral artery and vein for blood sampling and drug administration, respectively. Both cannulas were subcutaneously led to the back of the head and fixated in the neck with a rubber ring. Subsequently, the animals were chronically instrumented with two CMA/12 microdialysis guides (CMA/Microdialysis AB, Stockholm, Sweden) in different combinations of ST, for sampling in brain ECF, and LV and/or CM for sampling in CSF (ST + LV, ST + CM or LV + CM). For ST, the position of the microdialysis guide is: 1.0 mm anterior, 3.0 mm lateral, 3.4 mm ventral, relative to bregma. For LV, the position of the microdialysis guide is: 0.9 mm posterior, 1.6 mm lateral, 2.9 mm ventral, relative to the bregma. For CM, the position of the microdialysis guide is: 1.93 mm posterior, 3.15 mm lateral, 8.1 mm ventral, at an angle of 25° from the dorsoventral axis (towards anterior) and 18° lateral from the anteroposterior axis relative to lambda. The microdialysis guides were secured to the skull with 3 anchor screws and dental cement.

After the surgery the animals received 0.03 ml Temgesic^®^ intramuscularly (Schering-Plough, Amstelveen, The Netherlands) and 0.3 ml Ampicillan^®^ (Alfasan B.V., Woerden, The Netherlands) subcutaneously. One day prior to the experiment, the microdialysis dummies were replaced by the microdialysis probes (CMA/12 Elite, Polyarylethersulfone, molecular weight cut-off 20 kDa, CMA/Microdialysis AB, Stockholm, Sweden, with a semi-permeable membrane length of 4 mm for ST, and 1 mm for LV and CM).

### Experimental set-up

All experiments were performed as described by Westerhout et al. [20], with some modifications. In short, the in vivo microdialysis probe recovery of quinidine was determined on the basis of reverse dialysis [35]. The microdialysis probes in ST, LV and CM were perfused with different concentrations of quinidine (50, 200 and 1000 ng/ml) in perfusion fluid. To evaluate the potential effect of co-administration of tariquidar on the in vivo recovery of quinidine, several animals received an intravenous infusion of 15 mg/kg in 5 % glucose solution (100 μl/min/kg for a period of 10 min) with an automated pump (Pump 22 Multiple Syringe Pump, Harvard Apparatus, Holliston, USA) 30 min prior to the start of the reverse dialysis experiment. Control animals received an intravenous infusion of vehicle (100 μl/min/kg for a period of 10 min).

The in vivo recovery is defined as the ratio of the concentration difference between the dialysate (C_dial_) and perfusion fluid (C_in_) over the concentration in the perfusion fluid (Eq. 1) [36].

1 $${\text{In}}\,{\text{vivo}}\,{\text{recovery}} = \frac{{C_{in} - C_{dial} }}{{C_{in} }} $$

For the brain disposition experiments, the rats first received an intravenous infusion of 15 mg/kg tariquidar in 5 % glucose solution or vehicle 30 min prior to the administration of 10 or 20 mg/kg quinidine in saline (100 μl/min/kg for a period of 10 min). The start and duration of the infusion was corrected for internal volume of the tubing so that infusion started at t = 0 min. 10 min interval samples were collected between t = −1 h to t = 4 h, followed by 20 min interval samples from t = 4–6 h. After weighing the microdialysis vials they were stored at −80 °C before analysis.

For the determination of quinidine plasma concentrations, blood samples of 100 μl were taken, in parallel to the microdialysate samples, from the arterial cannula at t = −5 (blank), 2, 7, 10, 12, 17, 30, 60, 140, 240, and 360 min. All blood samples were temporarily stored in heparin (10 IU) coated Eppendorf cups before being centrifuged for 15 min at 5,000 rpm. The plasma was then pipetted into clean Eppendorf cups and stored at −20 °C before analysis.

At the end of the experiments the animals were sacrificed with an overdose of Nembutal (Ceva Sante Animale, Libourne, France). The animals were then perfused and decapitated to isolate the brain. After cleaning with saline, weighing, and freezing in liquid nitrogen, the brain was stored at −80 °C before analysis.

### Plasma protein binding

For the determination of plasma protein binding of quinidine, blood samples of 300 μl were taken at t = −30 (blank) and 60 min (with a concentration assumed to be approximately 1/2 × C_max_ [37]). After the blood sample at t = 360 min, an additional dose of 10 or 20 mg/kg in 10 min was given to be able to determine plasma protein binding at C_max_ (at t = 370 min). All blood samples were temporarily stored in heparin (10 IU) coated Eppendorf cups. The blank blood samples were spiked with quinidine to obtain a blood concentration of 100 ng/ml for the 10 mg/kg dose group and 200 ng/ml for the 20 mg/kg dose group. The spiked blood samples were then incubated in a shaking water bath at 37 °C for 30 min. All blood samples were centrifuged for 15 min at 5,000 rpm and the plasma was pipetted into clean Eppendorf cups and stored at −20 °C before analysis.

Plasma protein binding was determined with Centrifree^®^ ultrafiltration devices (Millipore BV, Etten-Leur, the Netherlands). All procedures were performed according to the user’s manual. The ultrafiltrate was diluted ten times with saline before the analysis.

### Concentration analysis

Quinidine concentrations in plasma, plasma ultrafiltrate, microdialysate, and total brain were determined as described by Syvänen et al. [32], using high pressure liquid chromatography (HPLC) with fluorescence detection. In short, to 20 μl of plasma, 50 μl internal standard (IS; 500 ng/ml quinine) was added. After homogenization with 200 μl borate buffer pH 10, 5 ml of methyl tert-butyl ether was added. After vortexing, centrifugation, and freezing of the aqueous layer, the organic phase was evaporated to dryness. The extracts were reconstituted in 100 μl of mobile phase and centrifuged at 4,000×*g* during 5 min. The clean plasma extracts were injected using a mobile phase with an acetonitrile/buffer ratio of 1:6.

To 20 μl of the plasma ultrafiltrate or microdialysate samples 20 μl IS was added, followed by vortexing before being directly injected into the HPLC system. Quinidine concentration in brain tissue was analyzed by the following steps: whole brain was homogenized in 50 mM phosphate buffer at pH 7.4. To 600 μl of the homogenate 100 μl IS and 100 μl 1 M sodium hydroxide was added. 5 ml methyl tert-butyl ether was then added, followed by vortexing and centrifugation. 4 ml of the supernatant was then transferred to a clean glass tube and 100 μl of 30 mM phosphoric acid was added. After vortexing and centrifugation, the supernatants were aspirated and discarded. The remaining aqueous phase was centrifuged for 10 min at 11,000×*g*. An aliquot of 50 μl was then transferred to clean glass vials and 20 μl was injected into the HPLC system.

Data acquisition and processing was performed using Empower^®^ data acquisition software (Waters, Etten-Leur, The Netherlands). For constructing the calibration curve, linear regression analysis was applied using weight factor 1/(y)^2^. Data analysis, statistical analysis, and plotting were performed using Microsoft^®^ Office Excel 2003 (Microsoft Corporation, USA).

### PK data analysis

All plasma concentrations were converted to unbound plasma concentrations, by correction for plasma protein binding. All microdialysate concentrations from ST, LV and CM were converted into brain ECF concentrations (C_ECF_) or CSF concentrations (C_CSF_) by division of the dialysate concentrations by the average in vivo recovery as determined for each microdialysis probe location (Eq. 2).

2 $$C_{ECF} \,{\text{or}}\,C_{CSF} = \frac{{C_{dial} }}{{{\text{in}}\,{\text{vivo}}\,{\text{recovery}}}} $$

Areas under the curve from t = 0 to t = 360 min (AUC_0–360_) were calculated by the trapezoidal rule and tested for differences by single factor ANOVA. The population PK models were developed and fitted to the data by means of non-linear mixed-effects modeling using the NONMEM software package (version 6.2, Icon Development Solutions, Ellicott City, Maryland, USA) and analyzed using the statistical software package S-Plus^®^ for Windows (version 6.2 Professional, Insightful Corp., Seattle, USA).

The PK model for quinidine plasma and brain concentrations was based on the systems-based PK (SBPK) approach we have previously applied to investigate the exchange between brain ECF and CSF of acetaminophen [20]. For this approach, the volumes of the different brain compartments were fixed to their physiological volumes. The rat brain intracellular space and brain_ECF_ volume were assumed to be 1.44 ml [38] and 290 μl [39], respectively. With a total CSF volume of 300 μl in the rat [40], the volumes of the LV, third and fourth ventricles, CM and subarachnoid space were assumed to be 50 μl [41, 42], 50 μl [43], 17 μl [44, 45] and 180 μl [40, 43], respectively. The intra-brain distribution was restricted by the physiological flow paths of brain ECF, in which brain ECF flows towards the CSF compartments at a rate of 0.2 μl/min [39, 46], and CSF flows from LV, through the third and fourth ventricle, to the CM and subsequently to the subarachnoid space (cranial and spinal) and back into blood at a rate of 2.2 μl/min [47].

Structural model selections for both the blood and brain PK model were based on the likelihood ratio test (*p* < 0.01), diagnostic plots (observed concentrations vs. individual and population predicted concentrations, weighted residuals vs. predicted time and concentrations), parameter correlations and precision in parameter estimates. The inter-animal variability in PK parameters was assumed to be log normally distributed. The residual error, which accounts for unexplained variability (e.g. measurement and experimental error and model-misspecification), was best described with a proportional error model.

The validity of the PK models was investigated by means of a visual predictive check [48–50]. Using the final PK parameter estimates, 1,000 curves were simulated. Subsequently, the median and the 5th and 95th percentile of the predicted concentrations were calculated, which represent the 90 % prediction interval. These were then compared with the observations.

In order to test the ruggedness of the model and estimate the precision of the parameters *n* = 100 non-parametric (case resampling) bootstraps were performed. To create the bootstrapped datasets, specific rat data (plasma and microdialysate concentrations) were removed randomly from the datasets and replaced with randomly selected rat data from the complete original dataset. Each of these permutations of the original dataset were fitted with the final model determined based on the original dataset. This results in a series of model fits, each with its own set of parameters. These results were displayed graphically and the descriptive statistics of the parameters were compared to parameter estimates of the final model. Only bootstrap runs that successfully minimized were used in this analysis.

## Results

All results are presented as average values ± standard error of the mean, unless stated otherwise.

### Quinidine PK

**Fig. 1:**
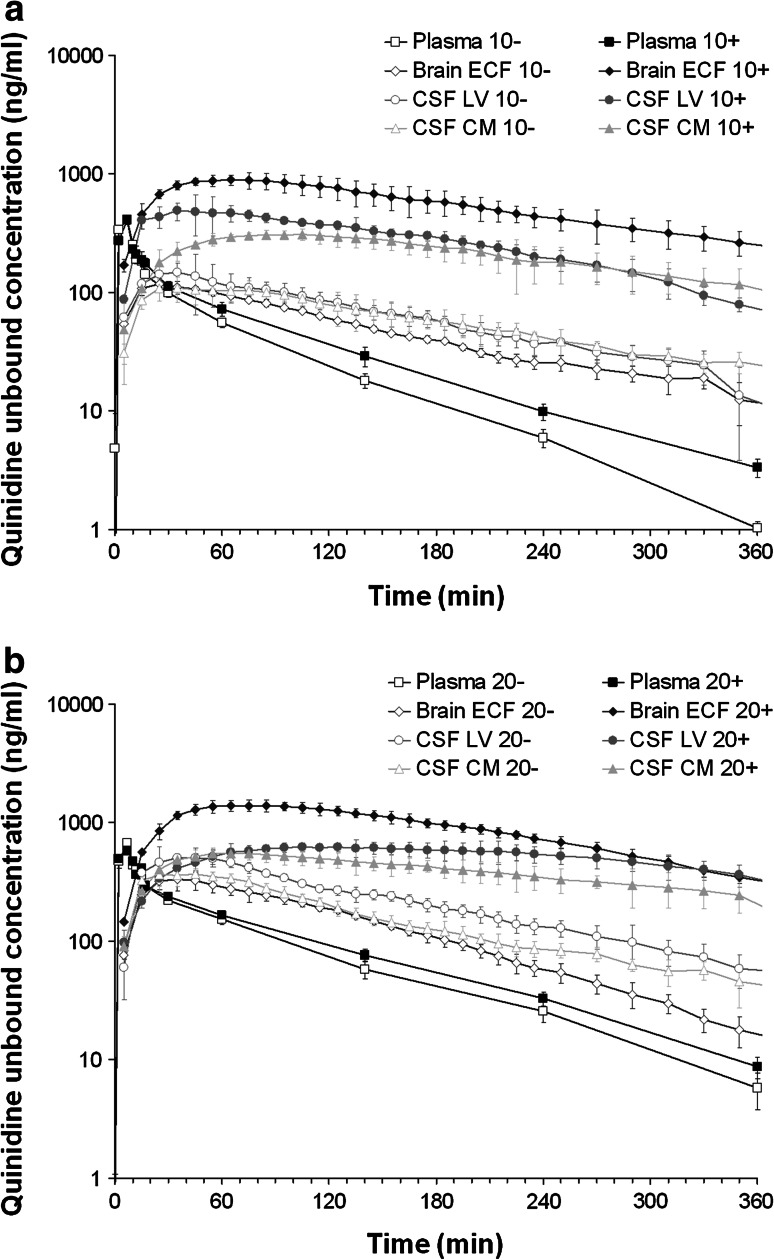
Average (geometric mean ± SEM) unbound quinidine concentration–time profiles following intravenous administration of quinidine, with (+) or without (−) co-administration of tariquidar (15/mg/kg). **a** 10 mg/kg quinidine dose: for plasma (*n* = 11 (−) and 6 (+)), brain_ECF_ (*n* = 6 (−) and 4 (+)), CSF_LV_ (*n* = 4 (−) and 3 (+)) and CSF_CM_ (*n* = 4 (−) and 4 (+). **b** 20 mg/kg quinidine dose. Plasma (*n* = 9 (−) and 11 (+)), brain_ECF_ (*n* = 5 (−) and 6 (+)), CSF_LV_ (*n* = 4 (−) and 4 (+)) and CSF_CM_ (*n* = 6 (−) and 6 (+))

The average unbound plasma (plasma_u_) and unbound brain (brain_u_) quinidine concentrations following the 10 and 20 mg/kg dose with or without co-administration of tariquidar are shown in Fig. 1. Plasma protein binding of quinidine was linear at an extent of 86.5 ± 5.5 %. It was not affected by co-administration of tariquidar. The co-administration of tariquidar slightly reduced the plasma elimination rate of plasma_u_ for both 10 and 20 mg/kg dose of quinidine. Data obtained by microdialysis from the brain_ECF_, LV (CSF_LV_) and CM (CSF_CM_) were corrected for in vivo recovery. The average in vivo recoveries for the brain_u_ concentrations in ST, LV and CM probes were not influenced by co-administration of tariquidar and were determined to be 9.1 ± 0.5, 2.9 ± 0.5 and 3.5 ± 0.5 %, respectively.

It can be seen that a higher dose of quinidine leads to higher brain_u_ quinidine concentrations in all brain compartments, but not to the same extent. Tariquidar increased all brain_u_ quinidine concentrations significantly (*p* < 0.01), but most pronouncedly for brain_ECF_. The effect of tariquidar was dependent on the quinidine dose; at the higher dose of quinidine, the increase in brain_u_ quinidine concentrations was less profound, as can be seen by the average brain_u_-to-plasma_u_ AUC_0–360_ ratios (Table 1). However, the difference between the brain_u_-to-plasma_u_ AUC_0–360_ ratios for the 10 and 20 mg/kg dose with co-administration of tariquidar was only significant (*p* < 0.05) for brain_ECF_. The relationship between brain_ECF_-to-CSF concentrations ratios was also very much dependent on tariquidar, and on average were increased from 0.77 ± 0.19 to 2.41 ± 0.56 and from 0.67 ± 0.21 to 2.02 ± 0.52, for the 10 and 20 mg/kg dose, respectively (Table 2). Significant differences in AUC ratios and concentrations between brain_ECF_ and CSF (either from LV or CM) were only observed for the groups that received the co-administration of tariquidar.

**Table 1:** Brain_u_-to-plasma_u_ AUC_0–360_ ratios for brain ECF, CSF_LV_ and CSF_CM_ for the 10 and 20 mg/kg dose without (−) and with (+) co-administration of tariquidar

Brain_u_-to-plasma_u_ AUC_0–360_ ratios	10^−^ (%)	10^+^ (%)	20^−^ (%)	20^+^ (%)
Brain_ECF_	135 ± 17	1,265 ± 213**^,‡^	150 ± 16^‡^	864 ± 64**^,‡†^
CSF_LV_	177 ± 39	624 ± 41**	257 ± 24	498 ± 74**
CSF_CM_	167 ± 16	479 ± 76**	184 ± 15	383 ± 33**

** Significantly (*p* < 0.01) different from the group without co-administration of tariquidar

^‡^Significantly (*p* < 0.05) different from the CSF-to-plasma_u_ AUC_0–360_ ratios

^†^Significantly (*p* < 0.05) different from the 10 mg/kg dose group with co-administration of tariquidar

**Table 2:** Brain_ECF_-to-CSF concentration ratios for the 10 and 20 mg/kg dose without (−) and with (+) co-administration of tariquidar

Brain_ECF_-to-CSF concentration ratios	10^−^	10^+^	20^−^	20^+^
Brain_ECF_-to-CSF_LV_	0.75 ± 0.09*	2.13 ± 0.47*	0.56 ± 0.18*	1.81 ± 0.57
Brain_ECF_-to-CSF_CM_	0.79 ± 0.25	2.70 ± 0.51*	0.78 ± 0.17	2.23 ± 0.37*
Brain_ECF_-to-CSF_average_	0.77 ± 0.19	2.41 ± 0.56*	0.67 ± 0.21	2.02 ± 0.52*

* Significantly (*p* < 0.05) different from 1

Also, end-of-experiment total brain concentrations (brain_total_) were obtained. These data were corrected for corresponding brain_ECF_ concentrations to represent deep brain (brain_deep_) concentrations. The brain_deep_ concentrations were determined to be on average 3.6 ± 1.6-fold higher than final brain_ECF_ concentrations for the control group and 6.3 ± 1.5-fold higher for the animals that received a co-administration of tariquidar (not significantly different). This indicates that P-gp also influences brain intracellular exposure.

### Compartmental modeling approach

All data were subjected first to compartmental PK analysis. It was shown that the plasma concentrations were best described by a three compartment model with inter-compartmental clearance (Q), and elimination clearance from the central compartment (CL_E_). The effect of the co-administration of tariquidar on the elimination clearance was found to be significant (*p* < 0.01, OF value reduction of 6.63 units).

**Fig. 2:**
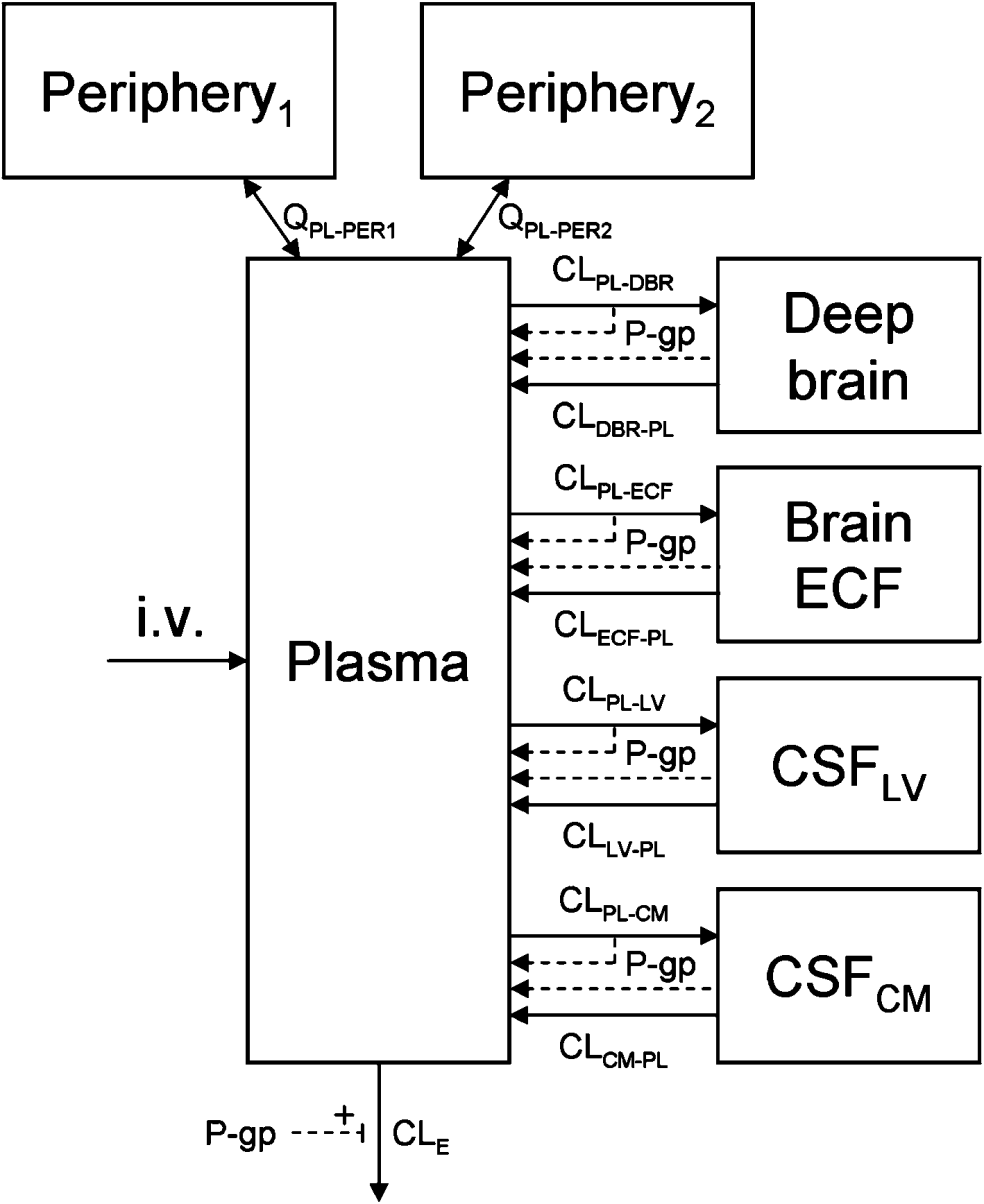
Diagram of the compartmental model that was used to describe the brain distribution of quinidine in the rat. CL_E_ is the elimination clearance from plasma, Q_PL–PERx_ is the inter-compartmental clearance between plasma and the first (x = 1) or second (x = 2) peripheral compartment. Further, for transfer clearances between compartments (CL_from comp-to comp_), denotations of the compartments are: *PL* plasma; *ECF* brain_ECF_; *DBR* brain_deep_; *LV* lateral ventricle; and *CM* cisterna magna. For peripheral and plasma compartments, *V* volume of distribution; for brain compartments, *V* physiological volume, not being shown in the model

To describe the concentrations in each of the brain compartments, four brain compartments were added (brain_ECF_, CSF_LV_, CSF_CM_ and brain_deep_). Drug transport between the plasma and the different brain compartments was determined by a transfer clearance between plasma and each of the brain compartments (CL_PL–BR_) and vice versa (CL_BR–PL_). In this model (Fig. 2) it was not possible to include drug transport between the different brain compartments because each brain compartment then has multiple routes of entry. The model was not able to identify the specific contribution of each route, resulting in transfer clearance value estimations near 0, which is not realistic. Therefore, we decided to remove the transport between the different brain compartments.

#### Distinction between passive and active transport clearances

The effect of P-gp on the different transfer clearances between plasma and the brain compartments was determined by comparing the parameter estimations for the rats that did to those rats that did not receive the co-administration of tariquidar. Thus, a distinction could be made between the passive and the active component of the transfer clearances.

The data were best described by a model in which P-gp reduced the transfer clearance from plasma to the brain compartments (i.e. influx hindrance) and increased the transfer clearance from the brain compartments to plasma (i.e. efflux enhancement). The transfer clearances between plasma and the different brain compartments that could be assigned to P-gp were incorporated into the model as previously described by Syvänen et al. [51]:

3 $$CL_{PL - BR} = CL_{PL - BR,p} - CL_{{PL - BR,P{\text {-}}gp}} $$

4 $$CL_{BR - PL} = CL_{BR - PL,p} - CL_{{BR - PL,P{\text {-}}gp}} $$

where the subscript ‘p’ denotes passive transport and ‘P-gp’ denotes P-gp-mediated transport.

#### Modeling quinidine concentration-dependent P-gp-mediated transport

Since P-gp-mediated transport is an active (saturable) process we have also tried to identify the maximal transport rate (T_m_) and the blood- or brain concentration for half-maximal transport (K_m_) as follows:

5 $$CL_{{PL - BR,P{\text {-}}gp}} = \frac{{T_{m,PL - BR} }}{{K_{m,PL - BR} + C_{PL,u} }} $$

6 $$CL_{{BR - PL,P{\text {-}}gp}} = \frac{{T_{m,BR - PL} }}{{K_{m,BR - PL} + C_{BR} }} $$

where C_PL,u_ is the unbound plasma concentration and C_BR_ is the concentration in one of the brain compartments. The parameter estimations of T_m_ and K_m_ resulted in high values for both T_m_ and K_m_ (results not shown), indicating that the plasma and brain concentrations in this study are not sufficiently high for saturating P-gp-mediated transport. The parameter estimations of T_m_ and K_m_ also resulted in too large coefficients of variation. Thus, our data were insufficient to determine the values of these parameters, and for the next modeling steps, P-gp-mediated transport had to be incorporated by means of a single clearance value, rather than by T_m_ and K_m_.

#### Modeling deep brain concentrations

Brain_deep_ concentrations were determined for samples obtained at the end-of-experiment time point. Based on previous studies in our lab with male Wistar WU rats (unpublished results), it was found that the brain_deep_-to-brain_ECF_ concentration ratio of quinidine was constant throughout the entire experimental period. We used this information to estimate brain_deep_ concentrations during the experiment.

#### Final compartmental model

**Fig. 3:**
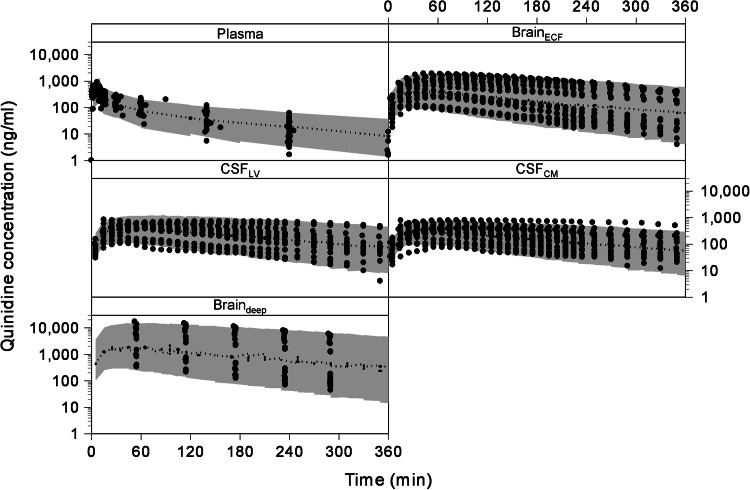
The visual predictive check of the compartmental model. The *dots* represent the individual data points and the *gray area* represents the 95 % prediction confidence interval. The different *boxes* represent the plasma, brain_ECF_, CSF_LV_, CSF_CM_ and brain_deep_ data

The final estimation of the PK parameters of the compartmental model is summarized in Table 3. The visual predictive check of the final compartmental model is given in Fig. 3. It can be seen that the compartmental model describes the data very well within the 95 % prediction interval, and also can cope with the large inter-individual variation as observed in the different brain concentrations. The goodness of fit plots of the plasma, brain_ECF_, CSF_LV_, CSF_CM_ and brain_ECF_ data with the compartmental model are available as supplemental material.

**Table 3:** Final estimation of the rat PK parameters for the compartmental model (±standard error)

Parameter	Value
CL_E_	158 ± 11 ml/min
P-gp effect on CL_E_	1.2 ± 0.1-fold increase
Q_PL–PER1_	822 ± 95 ml/min
Q_PL–PER2_	171 ± 28 ml/min
CL_PL–DBR,p_	1,430 ± 188 μl/min
CL_PL–DBR,P-gp_	1,270 ± 165 μl/min
CL_DBR–PL,p_	16.1 ± 1.3 μl/min
CL_DBR–PL,P-gp_	17.3 ± 2.4 μl/min
CL_PL–ECF,p_	36.6 ± 3.9 μl/min
CL_PL–ECF,P-gp_	25.8 ± 3.7 μl/min
CL_ECF–PL,p_	3.2 ± 0.2 μl/min
CL_ECF–PL,P-gp_	4.4 ± 0.7 μl/min
CL_PL–LV,p_	3.4 ± 0.7 μl/min
CL_PL–LV,P-gp_	1.1 ± 0.3 μl/min
CL_LV–PL,p_	0.4 ± 0.09 μl/min
CL_LV–PL,P-gp_	0.5 ± 0.2 μl/min
CL_PL–CM,p_	0.7 ± 0.08 μl/min
CL_PL–CM,P-gp_	0.07 ± 0.02 μl/min
CL_CM–PL,p_	0.1 ± 0.02 μl/min
CL_CM–PL,P-gp_	0.2 ± 0.06 μl/min
V_PL_	*10.6* *ml* [52]
V_PER1_	5.9 ± 0.5 l
V_PER2_	11.7 ± 1.6 l
V_DBR_	*1.44* *ml* [38]
V_ECF_	*290* *μl* [39]
V_LV_	*50* *μl* [41, 42]
V_CM_	*17* *μl* [44, 45]
η_CLE_	0.08 ± 0.02
ε_PL_	0.13 ± 0.02
ε_DBR_	0.06 ± 0.01
ε_ECF_	0.05 ± 0.01
ε_LV_	0.09 ± 0.02
ε_CM_	0.07 ± 0.01

Parameter values in italic are derived from literature. CL_E_ is the elimination clearance from plasma, Q_PL–PERx_ is the inter-compartmental clearance between plasma and the first (x = 1) or second (x = 2) peripheral compartment. Further, for transfer clearances between compartments (CL_from comp-to comp_), denotations of the compartments are: *PL* plasma; *ECF* brain_ECF_; *DBR* brain_deep_; *LV* lateral ventricle; and *CM* cistern magna. For peripheral and plasma compartments, *V* volume of distribution; for brain compartments, *V* volume. *η*_*i*_ inter-individual variability of parameter i; *ε*_*j*_ residual error on concentrations in compartment j. The additional subscripts ‘p’ and ‘P-gp’ denote passive transport and P-gp-mediated transport, respectively

### Systems-based modeling approach

As it was our goal to investigate the relationship between brain_ECF_ and CSF PK, we have applied a SBPK modeling approach. To more adequately describe CSF physiology, we have added two CSF compartments that represent the combined third and fourth ventricle (CSF_TFV_) and the subarachnoid space (CSF_SAS_), like we did previously for analysis of acetaminophen regional brain distribution [20]. Since we have no measurements of the concentrations in the third and fourth ventricle, the transfer clearance between plasma and third and fourth ventricle was assumed to be equal to the transfer clearance between plasma and LV.

#### Modeling CSF flow

In our first attempt of the SBPK approach the values of the brain ECF flow (Q_ECF_) and CSF flow (Q_CSF_) were fixed to their physiological values. However, it appeared that this value for Q_CSF_ was too high for proper description of quinidine concentration in the CSF compartments. Therefore the CSF production rate was estimated. To do so, the clearance from CSF_LV_ to plasma was fixed to 0, as otherwise Q_CSF_ was estimated to be 0. Thereby, the model was ‘forced’ to estimate the Q_CSF_, being 0.52 ± 0.25 μl/min. This value of Q_CSF_ was much lower than the physiological one (2.2 μl/min). An explanation for the reduced Q_CSF_ was searched for. It was found that quinidine is capable of inhibiting Na^+^-K^+^-ATPase activity [53], which is an enzyme at the apical membrane of the choroid plexus that leads to the formation of CSF [54, 55]. A potential influence of quinidine reducing CSF flow was investigated by a CSF quinidine concentration (C_CSF_)-dependent inhibition of Q_CSF_ by means of an E_max_ model (Eq. 7), in which Q_CSF,EF_ was the effective CSF flow.

7 $$Q_{CSF,EF} = Q_{CSF} \left( {1 - \frac{{C_{CSF} }}{{C_{CSF} + IC_{50} }}} \right) $$

The resulting estimated IC_50_ of quinidine was 209 ± 66 ng/ml. This value was 143-fold lower than reported (~30 μg/ml, [53]) and not considered realistic. As an alternative, we needed to fix the Q_CSF_ to its physiological value and to define the rate of transfer of quinidine from blood to CSF_LV_ and vice versa being equal.

#### Modeling P-gp-mediated transport

P-gp has been well described as an efflux transporter at the BBB [23–26]. However, the mechanism by which P-gp can exert its effect could be by so-called efflux enhancement or influx hindrance or both. The data were best described by the model with P-gp function solely as influx hindrance or combined influx hindrance and efflux enhancement. The observation that in vivo probe recovery of quinidine was not affected by tariquidar would be an indication that quinidine is transported by P-gp only via the influx hindrance mechanism [28, 56, 57]. However, as the largest reduction in the objective function value in the model was observed for a combined influx hindrance and efflux enhancement, this indicates that this model is most probably the best.

Based on the suggestion that P-gp functions as an influx transporter at the BCSFB [22, 27], the effect of P-gp on the clearance values between plasma and CSF was described as such. However, with our data we could not identify P-gp influx at the BCSFB. Therefore, we have tested models in which P-gp was considered to be an efflux transporter at the BCSFB or not present at the BCSFB at all. The data were best described by a model with P-gp as an efflux transporter at the BCSFB for LV, whereas it was absent for CM.

Again, we have tried to identify T_m_ and K_m_ values for P-gp-mediated transport of the SBPK model, but without success (results not shown). Therefore, P-gp-mediated transport had to be incorporated by means of a single clearance value, rather than by T_m_ and K_m_.

#### Modeling deep brain concentrations

Our assumption was that compounds, after passing the BBB and BCSFB would first enter brain_ECF_, before reaching the brain_deep_ compartment. However, since the brain_deep_ concentrations are much higher than the brain_ECF_ concentrations, and the physiological volume of the brain_deep_ compartment is much larger than the brain_ECF_ compartment, the mass transfer of quinidine from plasma, via the brain_ECF_ compartment, to the deep brain needs to be quite substantial. This route did not result in a model that could adequately describe the data. In contrast, a direct mass transfer from plasma into the brain_deep_ compartment did. Actually, the direct route through lipid membranes seems a rather plausible explanation for a lipophilic drug like quinidine, which has a logP of 2.36 in its neutral form [58].

#### Final SBPK model

**Fig. 4:**
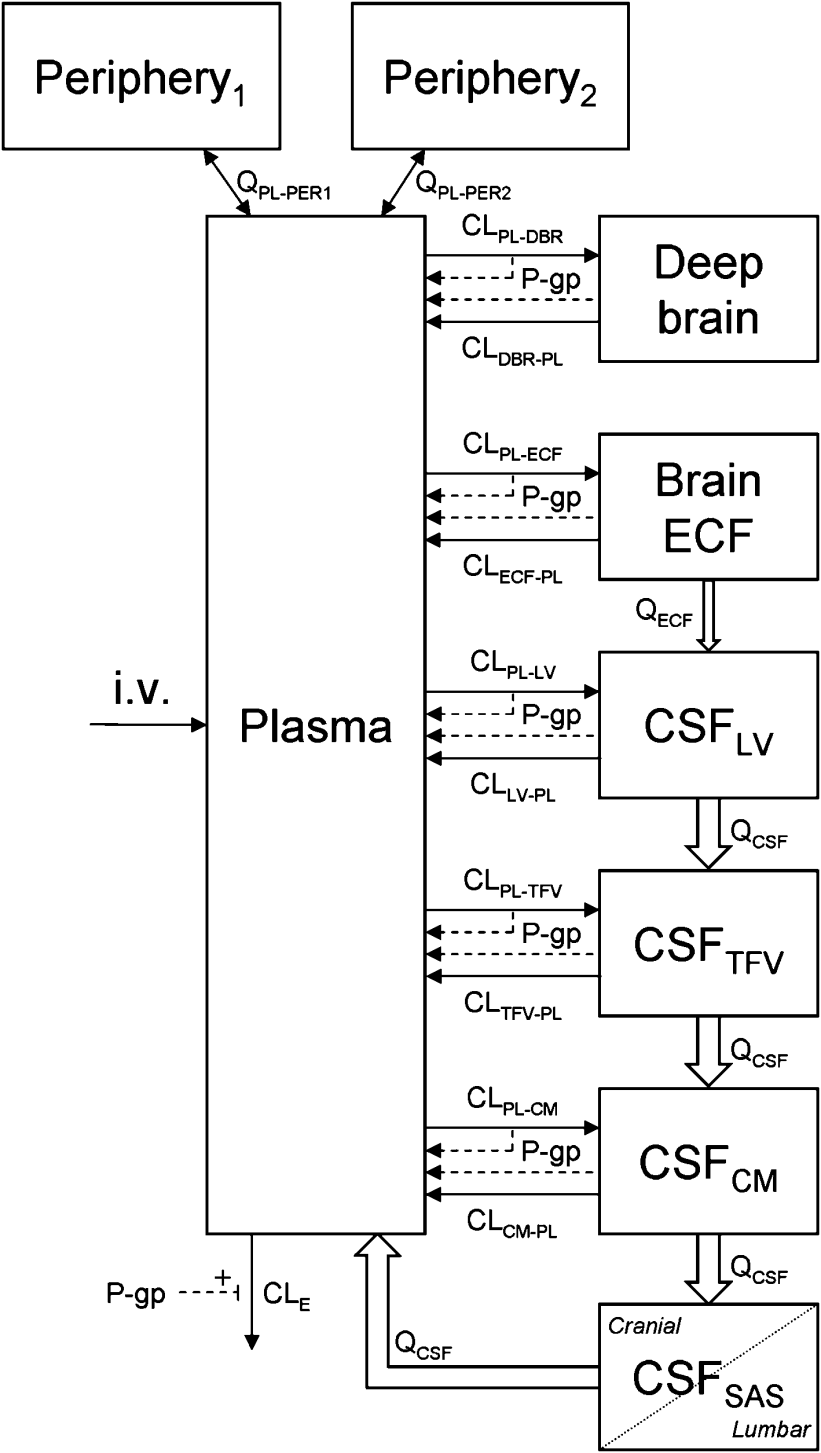
Diagram of the SBPK model that was used to describe the intra-brain distribution in the rat. CL_E_ is the elimination clearance from plasma, Q_PL–PERx_ is the inter-compartmental clearance between plasma and the first (x = 1) or second (x = 2) peripheral compartment. Further, for transfer clearances between compartments (CL_from comp-to comp_), denotations of the compartments are: *PL* plasma; *ECF* brain_ECF_; *DBR* brain_deep_; *LV* lateral ventricle; *TFV* third and fourth ventricle; *CM* cisterna magna and *SAS* subarachnoid space. Q_ECF_ is the flow rate of brain ECF, Q_CSF_ is the flow rate of CSF. For peripheral and plasma compartments, *V* volume of distribution; for brain compartments, *V* volume, not shown in the diagram

The final SBPK model is shown in Fig. 4. The differential equations of this model can be found in the appendix. The final estimation of the PK parameters is summarized in Table 4. Here, the parameters are the same as for Table 3, with the addition of the following: CL_PL–TFV_ is the clearance from plasma to CSF_TFV_, CL_TFV–PL_ is the clearance from CSF_TFV_ to plasma, Q_ECF_ is the flow rate of brain ECF, Q_CSF_ is the flow rate of CSF, V_TFV_ is the volume of the third and fourth ventricle combined and V_SAS_ is the volume of the subarachnoid space.

**Table 4:** Final estimation of the rat PK parameters for the different SBPK models (±standard error)

Parameter	Efflux enhancement	Influx hindrance	Efflux enhancement and influx hindrance
Objective function value	18,105	18,030	17,969
CL_E_	81.6 ± 11.4 ml/min	87.4 ± 10.5 ml/min	95.9 ± 11.0 ml/min
P-gp effect on CL_E_	1.9 ± 0.2-fold increase	2.1 ± 0.3-fold increase	1.9 ± 0.2-fold increase
Q_PL–PER1_	1,520 ± 177 ml/min	1,150 ± 138 ml/min	1,190 ± 135 ml/min
Q_PL–PER2_	84.2 ± 57.6 ml/min	360 ± 105 ml/min	333 ± 94 ml/min
CL_PL–DBR,p_	1,540 ± 182 μl/min	2,670 ± 501 μl/min	2,180 ± 384 μl/min
CL_PL–DBR,P-gp_	NA	2,430 ± 466 μl/min	1,900 ± 373 μl/min
CL_DBR–PL,p_	17.8 ± 1.5 μl/min	48.5 ± 9.6 μl/min	37.2 ± 7.2 μl/min
CL_DBR–PL,P-gp_	253 ± 40.4 μl/min	NA	19.6 ± 10.9 μl/min
CL_PL–ECF,p_	48.6 ± 6.3 μl/min	68.4 ± 9.1 μl/min	50.2 ± 5.0 μl/min
CL_PL–ECF,P-gp_	NA	54.8 ± 8.1 μl/min	33.8 ± 5.1 μl/min
CL_ECF–PL,p_	7.1 ± 1.2 μl/min	9.3 ± 1.4 μl/min	6.3 ± 0.8 μl/min
CL_ECF–PL,P-gp_	33.1 ± 8.1 μl/min	NA	5.3 ± 1.7 μl/min
CL_PL–LV,p_	7.2 ± 0.8 μl/min	8.4 ± 0.8 μl/min	9.0 ± 0.9 μl/min
CL_PL–LV,P-gp_	NA	3.0 ± 0.7 μl/min	3.8 ± 0.8 μl/min
CL_LV–PL,p_	0.03 ± 0.01 μl/min	0.04 ± 0.01 μl/min	0.04 ± 0.01 μl/min
CL_LV–PL,P-gp_	1.2 ± 0.4 μl/min	NA	0 μl/min
CL_PL–CM,p_	1.3 ± 0.3 μl/min	1.1 ± 0.3 μl/min	1.1 ± 0.3 μl/min
CL_PL–CM,P-gp_	NA	0 μl/min	0 μl/min
CL_CM–PL,p_	3.7 ± 0.5 μl/min	4.0 ± 0.5 μl/min	4.1 ± 0.5 μl/min
CL_CM–PL,P-gp_	0 μl/min	NA	0 μl/min
Q_ECF_	*0.2* *μl/min* [39, 46]	*0.2* *μl/min* [39, 46]	*0.2* *μl/min* [39, 46]
Q_CSF_	*2.2* *μl/min* [47]	*2.2* *μl/min* [47]	*2.2* *μl/min* [47]
V_PL_	*10.6* *ml* [52]	*10.6* *ml* [52]	*10.6* *ml* [52]
V_PER1_	13.2 ± 1.8 l	6.4 ± 1.6 l	6.8 ± 1.7 l
V_PER2_	5.8 ± 2.6 l	13.9 ± 2.0 l	13.3 ± 2.2 l
V_DBR_	*1.44* *ml* [38]	*1.44* *ml* [38]	*1.44* *ml* [38]
V_ECF_	*290* *μl* [39]	*290* *μl* [39]	*290* *μl* [39]
V_LV_	*50* *μl* [41, 42]	*50* *μl* [41, 42]	*50* *μl* [41, 42]
V_TFV_	50 μl [43]	50 μl [43]	50 μl [43]
V_CM_	*17* *μl* [44, 45]	*17* *μl* [44, 45]	*17* *μl* [44, 45]
V_SAS_	*180* *μl* [40, 43]	*180* *μl* [40, 43]	*180* *μl* [40, 43]
η_CL10_	0.20 ± 0.09	0.16 ± 0.08	0.14 ± 0.06
ε_PL_	0.29 ± 0.04	0.22 ± 0.02	0.22 ± 0.03
ε_DBR_	0.06 ± 0.01	0.07 ± 0.02	0.07 ± 0.02
ε_ECF_	0.07 ± 0.01	0.07 ± 0.01	0.06 ± 0.01
ε_LV_	0.10 ± 0.01	0.10 ± 0.01	0.11 ± 0.02
ε_CM_	0.06 ± 0.01	0.07 ± 0.01	0.08 ± 0.02

Parameter values in italic are derived from literature; NA implicates that the parameter is not available in the specific model

**Fig. 5:**
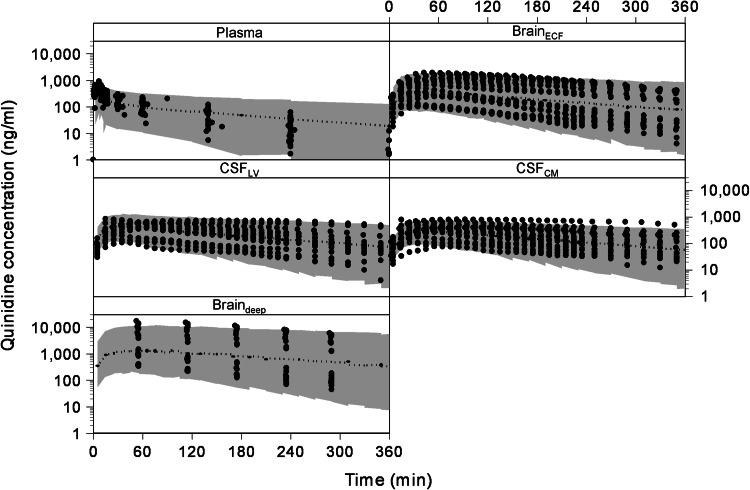
The visual predictive check of the final SBPK model. The *dots* represent the individual data points and the *gray area* represents the 95 % prediction confidence interval. The *x* axis represents the time (min) and the *y* axis represents the quinidine concentrations (ng/ml). The different *boxes* represent the plasma, brain ECF, CSF_LV_, CSF_CM_ and brain_deep_ data, respectively

The visual predictive check of the final model is given in Fig. 5. It can be seen that the final model describes the data very well within the 95 % prediction interval, and also can cope with the large inter-individual variation in brain concentrations. The goodness of fit plots of the plasma, brain_ECF_, CSF_LV_, CSF_CM_ and brain_ECF_ data with the final SBPK model are available as supplemental material.

## Discussion

In the development of CNS-targeted drugs, the prediction of human CNS target exposure is a big challenge. While CSF concentrations are often considered to be the best available surrogate for brain target site concentrations in humans, a generally applicable relationship between CSF concentrations and brain_ECF_ concentrations does not exist. [5, 12–14] Previous studies have indicated that, at steady-state conditions, CSF to brain_ECF_ concentration ratios were between threefold (either higher or lower) for compounds that freely diffuse across the BBB and BCSFB, while for compounds being brain barrier transporter substrates the difference may be higher [6–10]. Combining their data showed that 24 % (21/89) of the P-gp substrates had a CSF-to-brain_ECF_ concentration ratio larger than 3. Then, prediction of brain_ECF_ concentrations on the basis of CSF concentrations gets inadequate. This indicates that we need to improve our understanding of the impact of P-gp functionality at the brain barriers in order to be able to predict human CNS brain_ECF_ concentrations.

By using the multiple microdialysis probe approach [20], we investigated the direct relationships between brain ST concentrations and those in different CSF locations, and unbound plasma concentrations in the rat. We have focused on P-gp-mediated efflux transport functionality whereas it has been reported to function as an influx transporter at the BCSFB [22, 27]. This could have major implications for the relationship between CSF concentrations and brain ECF concentrations for compounds that are substrates for P-gp-mediated transport. To investigate the specific contribution of P-gp-mediated transport, quinidine was used as a paradigm P-gp substrate, with inhibition of P-gp by co-administration of tariquidar. Tariquidar is known to inhibit P-gp with a half-maximum inhibition constant (IC_50_) of approximately 25 ng/ml [59]. Previous work by Bankstahl et al. [60] and Syvänen et al. [61] have indicated that a 15 mg/kg dose of tariquidar results in plasma and brain concentrations over 50-fold higher than the IC_50_ value up to 3 h after administration. Therefore, it is plausible to assume that the dose of tariquidar is sufficient to fully inhibit P-gp throughout the entire experimental period. Advanced mathematical modelling was used to finally determine the interaction between systems physiology and quinidine. Our key findings indicated that: (1) brain_ECF_- and CSF-to-unbound plasma AUC_0–360_ ratios were all over 100 %, indicating influx transport by using unbound concentrations; (2) P-gp also restricts brain intracellular exposure; (3) a direct transport route of quinidine from plasma to brain cells exists; (4) P-gp-mediated efflux of quinidine at the BBB seems to result of combined efflux enhancement and influx hindrance; (5) P-gp at the BCSFB at the level of the LV functions as an efflux transporter or, at the CM, is not functioning at all.

In previous studies brain_ECF_ concentrations were estimated on the basis of total brain concentrations and the brain unbound fraction, determined by equilibrium dialysis of drug-spiked brain homogenates [6–8]. However, brain tissue homogenization destroys cell structures unmasking binding sites that are normally not accessible to a drug [9], potentially leading to underestimation of the in vivo brain unbound fraction. The use of the brain slice technique is an improvement [62]. Liu et al. [8], and Fridén et al. [10], have applied this technique to calculate the brain unbound fraction, being further used to estimate brain_ECF_ concentrations. Comparison of the brain homogenate method with brain slice technique indicated that the brain unbound fraction was over 50 % different for 5 out of 7 compounds [8]. Liu and colleagues [9] have later applied the microdialysis technique for direct measurement of unbound brain_ECF_ concentrations and compared those to CSF concentrations sampled at steady-state. They found that the ratio of CSF over brain_ECF_ concentrations was larger for 1 out of the 7 P-gp substrates.

To our surprise, we found that unbound concentrations in brain were significantly larger than unbound concentrations in plasma. This appears to be in contrast to previous studies by Liu et al. [9] and Kodaira et al. [63] in which unbound brain-to-unbound plasma (brain_u_/plasma_u_) concentration ratios at assumed steady state were well below unity. While our results were quite comparable to the results of Liu et al. [9] and Kodaira et al. [63], a substantial difference was found for the (calculated) unbound brain (ECF) concentrations between these studies, and ours. Liu et al. determined the brain free fraction with the brain homogenate method and found an unbound brain fraction comparable to the unbound brain fraction that was found by Kodaira et al. by the brain slice technique (3.6 and 2.4 %, respectively). In contrast, the unbound brain fraction in our study was calculated to be 28 % (brain_ECF_ concentration divided by the total brain concentration). However, Liu et al. reported a 3-fold difference in the brain_u_ concentrations when calculated on the basis of the brain homogenate free fraction, compared to using microdialysis data when corrected for in vitro recovery [9]. We measured both in vitro (33 %) and in vivo recovery (9 %), and found that the in vivo recovery was 3.5-fold lower. If we would calculate the brain_ECF_/plasma_u_ concentration ratio at maximal concentrations, like Liu et al. did, and assume that for Liu also a 3.5-fold lower in vivo recovery would apply, then the brain_ECF_/plasma_u_ concentration ratio would be comparable to ours.

For the brain_ECF_/plasma_u_ AUC_0–360_ ratios, we found values significantly larger than unity as in the elimination phase the rate of decline in plasma concentrations was larger than those observed in CSF and brain_ECF_. We cannot compare these findings with Kodaira and Liu because their studies did not include an elimination phase.

Therefore, based on our data, it appears that quinidine is also transported by other transporters at the BBB and BCSFB, in the direction of the brain. However, there is no direct information in literature to support this. We could only find the following potential contributions: Van Montfoort et al. [64] reported that quinidine is transported by OCTs. This observation was made in an in vitro study, and was found to occur for a pH of 6, but not significantly at pH 7.4. The question remains how this relates to the in vivo situation, like ours. Giacomini et al. [65] stated that quinidine is a potential inhibitor of OCTs. OCTs have been localized both at the BBB [66], as well as at the BCSFB [67]. Thus, the possibility of active influx transport for quinidine at the BBB and BCSFB remains to be further investigated. Alternatively, it could reflect a passive “ion-trapping” process, governed by lower pH values in brain ECF (pH ~7.3) than in plasma (pH ~7.4). However, as quinidine is a diprotic base with pKas 4.2 and 7.9 [30], the low difference between the % ionized at pH 7.3 (80 %) and pH 7.4 (76 %) does not seem to explain our findings.

According to the “smaller than threefold concentration ratio paradigm” [7], differences between brain_ECF_ and CSF concentrations of quinidine as found in this study (on average 0.72 ± 0.20) would be considered pharmacokinetically irrelevant. However, upon co-administration of tariquidar this ratio increased 3.1-fold (to the value of 2.22 ± 0.57). This means that P-gp functionality and variations thereof may have an important effect on the brain_ECF_–CSF ratio and the extrapolation from rats to humans, as is discussed by de Lange [68, 69]. However, quinidine is a strong P-gp substrate and it remains to be investigated what the impact of P-gp functionality on the brain_ECF_–CSF concentration relationships would be for weaker substrates.

Several different models for P-gp-mediated transport have been suggested. [28, 51, 56, 57, 70–72]. The first model is described as the “classical pump model” in which a P-gp substrate is transported from the cytosol to the extracellular space against a concentration gradient (so-called “efflux enhancement”). [51, 56, 70–72] The second model can be described as a “vacuum cleaner model” in which a lipophilic compound that is diffusing across the cellular membrane, is interacting with P-gp within the lipid bilayer of the cellular membrane and is then transported back into the extracellular space. [28, 70–72] The third model is described as the “flippase model” in which a lipophilic compound within the lipid bilayer at the cytosolic side is flipped to the extracellular side where it diffuses back into the extracellular space. [51, 56, 57, 70–72] In the second and third model P-gp prevents the entry of compounds to the brain by a process which is called “influx hindrance”. Based on the SBPK modeling results, it appears that for quinidine P-gp acts via combined influx hindrance and efflux enhancement. This is in line with the localization of P-gp at both the luminal and abluminal membrane of the BBB [21].

For the potential role of P-gp at the BCSFB, there have been some indications that P-gp could function as an influx transporter at the BCSFB [22, 27]. We anticipated this to be among our findings, however, with our data we could not identify P-gp influx at the level of the BCSFB. Instead, the results of the SBPK modelling suggest that P-gp at the BCSFB functions as an efflux transporter (LV) or is not functioning at all (CM).

Then, interestingly, the co-administration of tariquidar results in an increase of the total brain-to-brain_ECF_ concentration ratio, which has also been observed in an earlier study on quinidine at our lab by Syvänen et al. [32]. This indicates that P-gp is also located beyond the BBB at the parenchymal and perivascular astrocytes, which is in line with several reports [73–78].

In our current study we obtained in parallel brain ST, CSF and plasma concentration–time profiles, under dynamic conditions, included corrections for in vivo probe recoveries, and plasma protein binding to finally obtain unbound concentrations in these body compartments. It is anticipated that this approach, combined with advanced mathematical modelling, will further improve revealing the mechanisms underlying the relationship between brain_ECF_ and CSF concentrations than will steady-state and/or single end-of-experiment CSF concentrations [79]. Having information on concentration–time profiles following a single administration is relevant as we need time-dependent data to decipher the rate and extent of processes of drug transport into, within, and out of the brain [80]. It provides the best basis to further explore the multiple dose regimens as used in the clinic, for which it is know that a true steady state condition is actually not reached.

Finally, in striving towards reduction on the use of animals on one hand, and the fact that systematic studies on the inter-relationship of plasma PK, BBB transport and intra-brain distribution, cannot be performed in human, the use of the multiple microdialysis probe approach [20], obtaining a total of 84 samples per animal, results in a great reduction in the number of animals required for these type of studies compared to the single time point measurements.

## Conclusion

It is concluded that in parallel obtained data on unbound brain_ECF_, CSF and plasma concentrations, under dynamic conditions, combined with advanced mathematical modelling is a most valid approach to reveal the mechanisms underlying the relationship between brain_ECF_ and CSF concentrations, which is significantly influenced by activity of P-gp. This indicates that information on functionality of P-gp is important for the prediction of human brain target site concentrations of P-gp substrates on the basis of human CSF concentrations, and provides further guide to unravelling mechanisms and drug properties that govern the transport into, within, and out of the brain, for translational purposes.

### Electronic supplementary material

Below is the link to the electronic supplementary material.

Supplemental Fig. 1. The goodness of fit plot of the compartmental model for the plasma data Supplementary material 1 (TIFF 601 kb)

Supplemental Fig. 2. The goodness of fit plot of the compartmental model for the brain_ECF_ data Supplementary material 2 (TIFF 640 kb)

Supplemental Fig. 3. The goodness of fit plot of the compartmental model for the CSF_LV_ data Supplementary material 3 (TIFF 628 kb)

Supplemental Fig. 4. The goodness of fit plot of the compartmental model for the CSF_CM_ data Supplementary material 4 (TIFF 655 kb)

Supplemental Fig. 5. The goodness of fit plot of the compartmental model for the brain_deep_ data Supplementary material 5 (TIFF 582 kb)

Supplemental Fig. 6. The goodness of fit plot of the final SBPK model for the plasma data Supplementary material 6 (TIFF 602 kb)

Supplemental Fig. 7. The goodness of fit plot of the final SBPK model for the brain_ECF_ data Supplementary material 7 (TIFF 645 kb)

Supplemental Fig. 8. The goodness of fit plot of the final SBPK model for the CSF_LV_ data Supplementary material 8 (TIFF 638 kb)

Supplemental Fig. 9. The goodness of fit plot of the final SBPK model for the CSF_CM_ data Supplementary material 9 (TIFF 657 kb)

Supplemental Fig. 10. The goodness of fit plot of the final SBPK model for the brain_deep_ data Supplementary material 10 (TIFF 578 kb)

## Appendix

### Differential equations

The mass balance equations describing the final SBPK model were expressed as follows.

Plasma:

$$dA_{pl,u} /dt = dose - k_{PL - PER1} A_{pl,u} + k_{PER1 - PL} A_{PER1} - k_{PL - PER2} A_{pl,u} + k_{PER2 - PL} A_{PER2} - k_{PL - DBR} A_{pl,u} + k_{DBR - PL} A_{DBR} - k_{PL - ECF} A_{pl,u} + k_{ECF - PL} A_{ECF} - k_{PL - LV} A_{pl,u} + k_{LV - PL} A_{LV} - k_{PL - TFV} A_{pl,u} + k_{TFV - PL} A_{TFV} - k_{PL - CM} A_{pl,u} + k_{CM - PL} A_{CM} + (Q_{CSF} /V_{SAS} )A_{SAS} - k_{E} A_{PL,u}$$

$$C_{PL,u} = A_{PL,u} /V_{PL}$$

Periphery:

$$dA_{PER1} /dt = k_{PL - PER1} A_{PL,u} - k_{PER1 - PL} A_{PER1}$$

$$C_{PER1} = A_{PER1} /V_{PER1}$$

$$dA_{PER2} /dt = k_{PL - PER2} A_{PL,u} - k_{PER2 - PL} A_{PER2}$$

$$C_{PER2} = A_{PER2} /V_{PER2}$$

Brain_deep_:

$$dA_{DBR} /dt = k_{PL - DBR} A_{PL,u} - k_{DBR - PL} A_{DBR}$$

$$C_{DBR} = A_{DBR} /V_{DBR}$$

Brain_ECF_:

$$dA_{ECF} /dt = k_{PL - ECF} A_{PL,u} - k_{ECF - PL} A_{ECF} - (Q_{ECF} /V_{ECF} )A_{ECF}$$

$$C_{ECF} = A_{ECF} /V_{ECF}$$

CSF_LV_:

$$dA_{LV} /dt = k_{PL - LV} A_{PL,u} - k_{LV - PL} A_{LV} + (Q_{ECF} /V_{ECF} )A_{ECF} - (Q_{CSF} /V_{LV} )A_{LV}$$

$$C_{LV} = A_{LV} /V_{LV}$$

CSF_TFV_:

$$dA_{TFV} /dt = k_{PL - TFV} A_{PL,u} - k_{TFV - PL} A_{TFV} + (Q_{CSF} /V_{LV} )A_{LV} - (Q_{CSF} /V_{TFV} )A_{TFV}$$

$$C_{TFV} = A_{TFV} /V_{TFV}$$

CSF_CM_:

$$dA_{CM} /dt = k_{PL - CM} A_{PL,u} - k_{CM - PL} A_{CM} + (Q_{CSF} /V_{TFV} )A_{TFV} - (Q_{CSF} /V_{CM} )A_{CM}$$

$$C_{CM} = A_{CM} /V_{CM}$$

CSF_SAS_:

$$dA_{SAS} /dt = (Q_{CSF} /V_{CM} )A_{CM} - (Q_{CSF} /V_{SAS} )A_{SAS}$$

$$C_{SAS} = A_{SAS} /V_{SAS}$$

where $$k_{E} = (CL_{E,p} + CL_{{E,P{\text {-}}gp}} )/V_{PL}$$; $$k_{PL - PER1} = Q_{PL - PER1} /V_{PL}$$; $$k_{PER1 - PL} = Q_{PL - PER1} /V_{PER1}$$; $$k_{PL - PER2} = Q_{PL - PER2} /V_{PL}$$; $$k_{PER2 - PL} = Q_{PL - PER2} /V_{PER2}$$; $$k_{PL - DBR} = (CL_{PL - DBR,p} - CL_{{PL - DBR,P{\text {-}}gp}} )/V_{PL}$$; $$k_{DBR - PL} = (CL_{DBR - PL,p} + CL_{{DBR - PL,P{\text {-}}gp}} )/V_{DBR}$$; $$k_{PL - ECF} = (CL_{PL - ECF,p} - CL_{{PL - ECF,P{\text {-}}gp}} )/V_{PL}$$; $$k_{ECF - PL} = (CL_{ECF - PL,p} + CL_{{ECF - PL,P{\text {-}}gp}} )/V_{ECF}$$; $$k_{PL - LV} = (CL_{PL - LV,p} - CL_{PL - LV,P{\text {-}}gp} )/V_{PL}$$; $$k_{LV - PL} = (CL_{LV - PL,p} + CL_{{LV - PL,P{\text {-}}gp}} )/V_{LV}$$; $$k_{PL - TFV} = (CL_{PL - TFV,p} - CL_{{PL - TFV,P{\text {-}}gp}} )/V_{PL}$$; $$k_{TFV - PL} = (CL_{TFV - PL,p} + CL_{{TFV - PL,P{\text {-}}gp}} )/V_{TFV}$$; $$k_{PL - CM} = (CL_{PL - CM,p} - CL_{{PL - CM,P{\text {-}}gp}} )/V_{PL}$$; and $$k_{CM - PL} = (CL_{CM - PL,p} + CL_{{CM - PL,P{\text {-}}gp}} )/V_{CM} .$$

## Variables

A_i_: Amount of quinidine in compartment i (ng)
C_i_: Concentration of quinidine in compartment i (ng/ml)
K: Rate constant (min^−1^)
Q: Flow rate (ml/min)
CL: Clearance (ml/min)
V: Volume (ml)

## Subscripts

PL: Plasma
PL,u: Unbound quinidine in plasma
PERi: Peripheral compartment i
ECF: Brain ECF
CSF: CSF
LV: Lateral ventricle
TFV: Third and fourth ventricle
CM: Cisterna magna
SAS: Subarachnoid space
