# Supplementary figures and images for: The impact of P-gp functionality on non-steady state relationships between CSF and brain extracellular fluid
Source: J Pharmacokinet Pharmacodyn. 2013 Mar 29;40(3):327–42. doi: 10.1007/s10928-013-9314-4 (PMC4269305; doi:10.1007/s10928-013-9314-4)

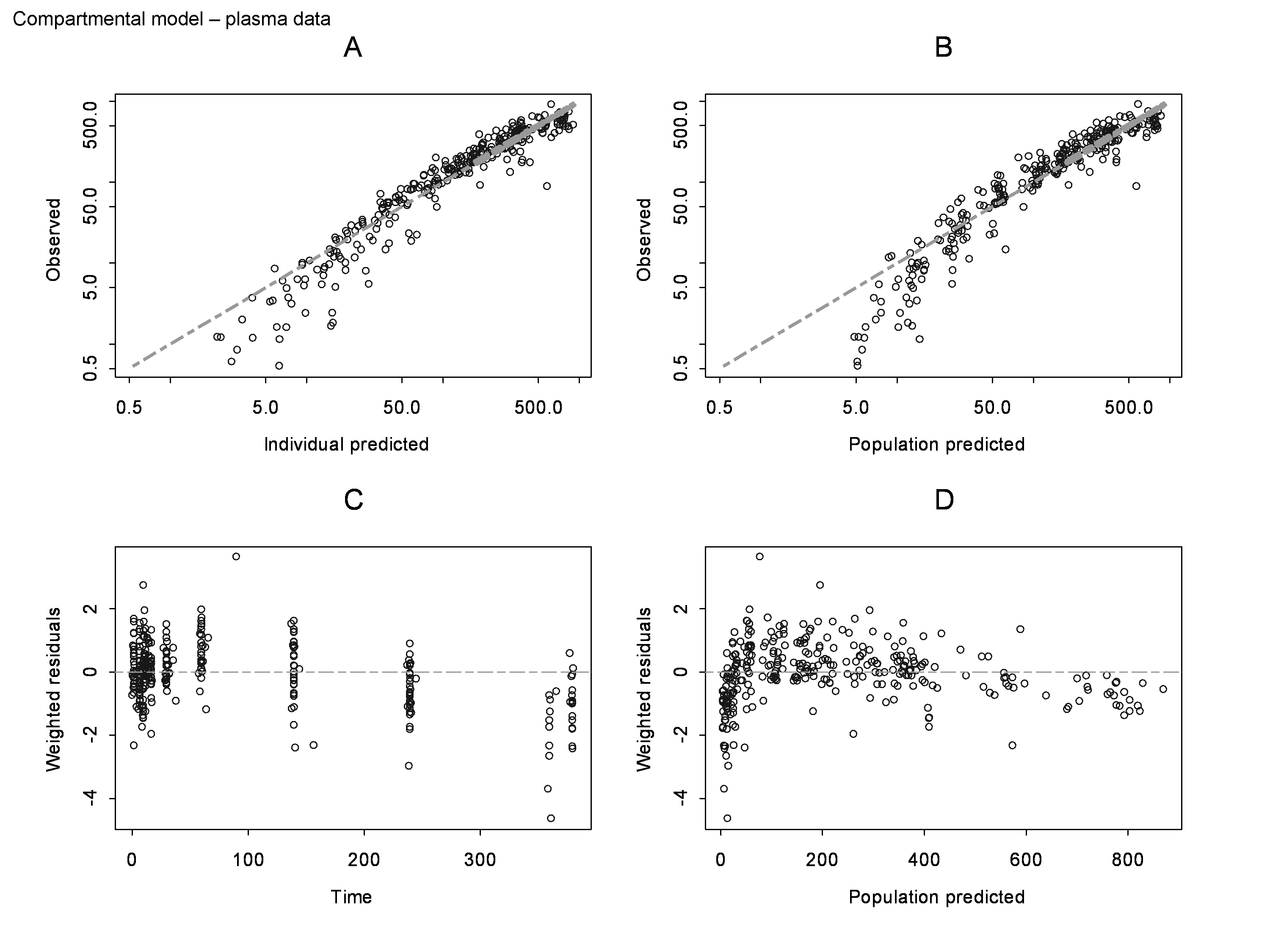

Supplement: Supplementary file 1 — Supplemental Fig. 1. The goodness of fit plot of the compartmental model for the plasma data Supplementary material 1 (TIFF 601 kb) [file 10928_2013_9314_MOESM1_ESM.tif]

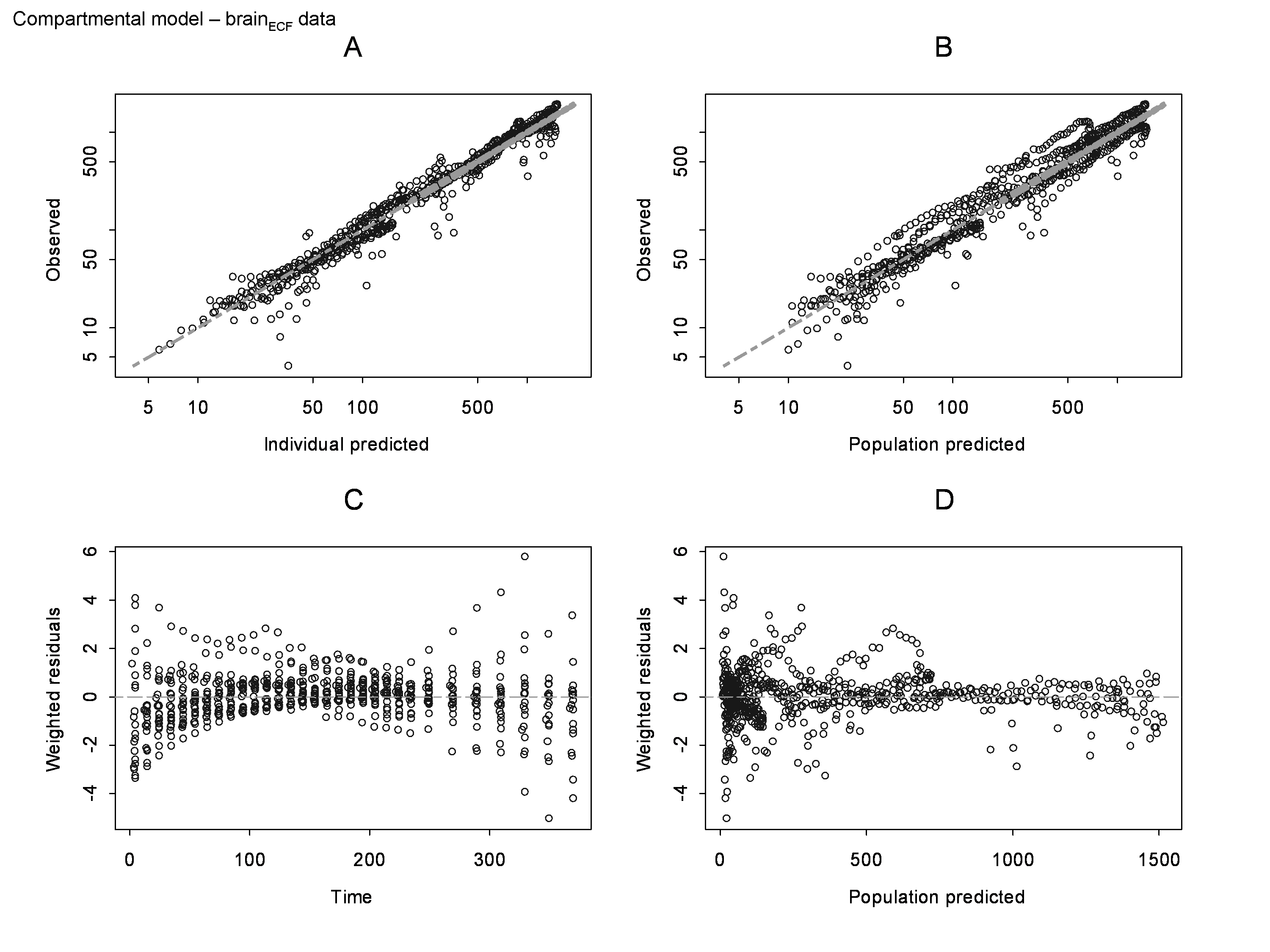

Supplement: Supplementary file 2 — Supplemental Fig. 2. The goodness of fit plot of the compartmental model for the brainECF data Supplementary material 2 (TIFF 640 kb) [file 10928_2013_9314_MOESM2_ESM.tif]

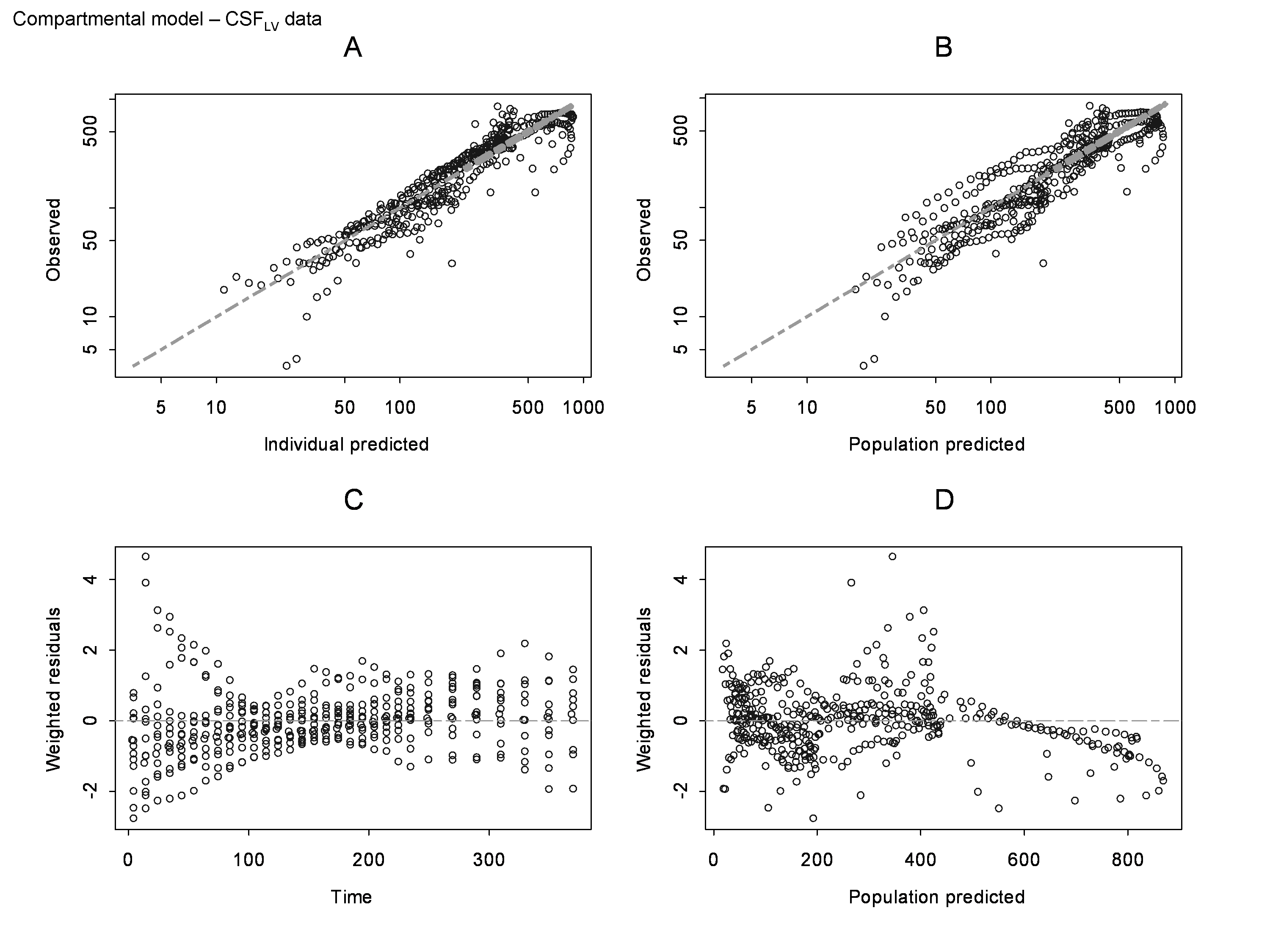

Supplement: Supplementary file 3 — Supplemental Fig. 3. The goodness of fit plot of the compartmental model for the CSFLV data Supplementary material 3 (TIFF 628 kb) [file 10928_2013_9314_MOESM3_ESM.tif]

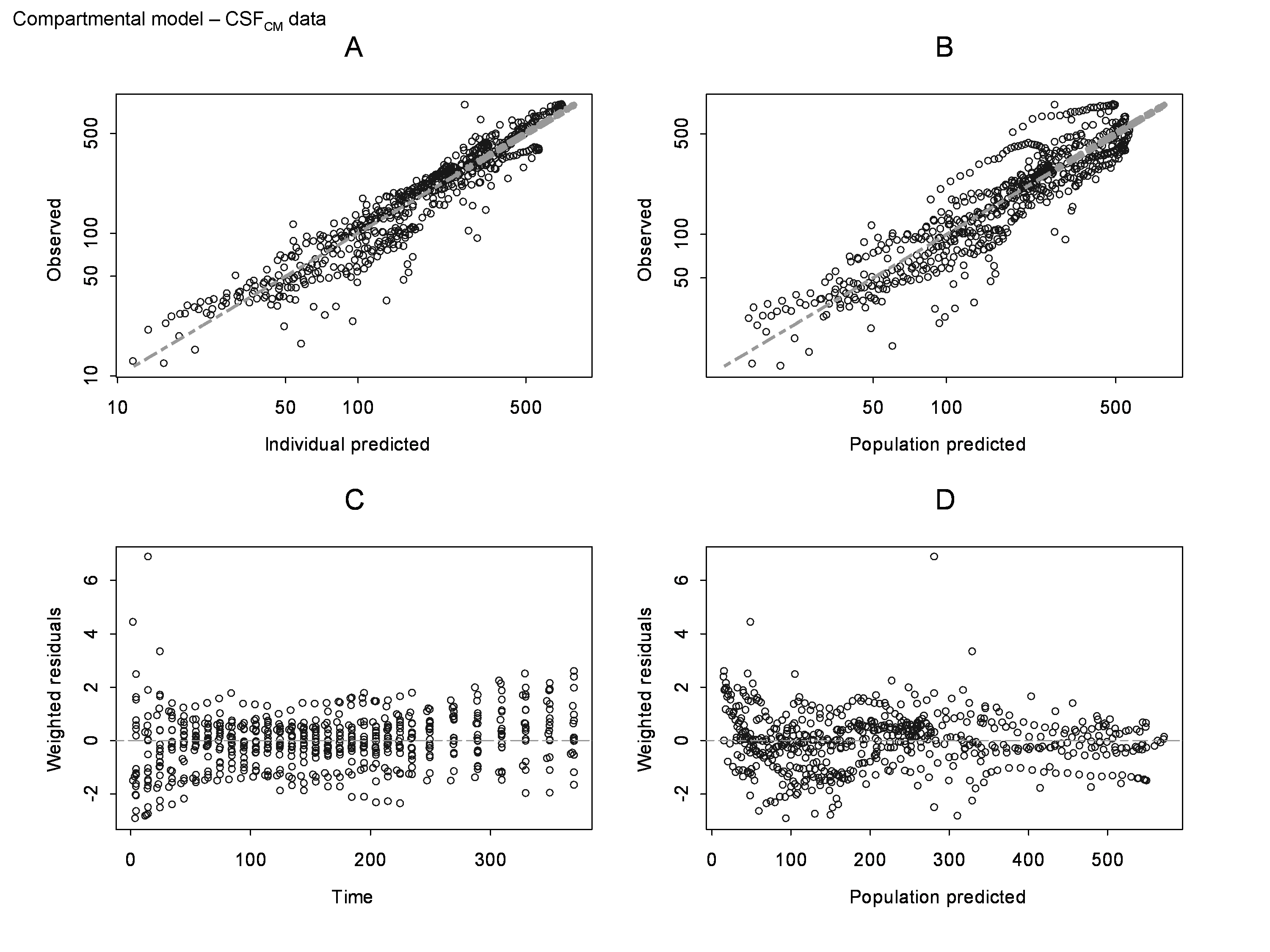

Supplement: Supplementary file 4 — Supplemental Fig. 4. The goodness of fit plot of the compartmental model for the CSFCM data Supplementary material 4 (TIFF 655 kb) [file 10928_2013_9314_MOESM4_ESM.tif]

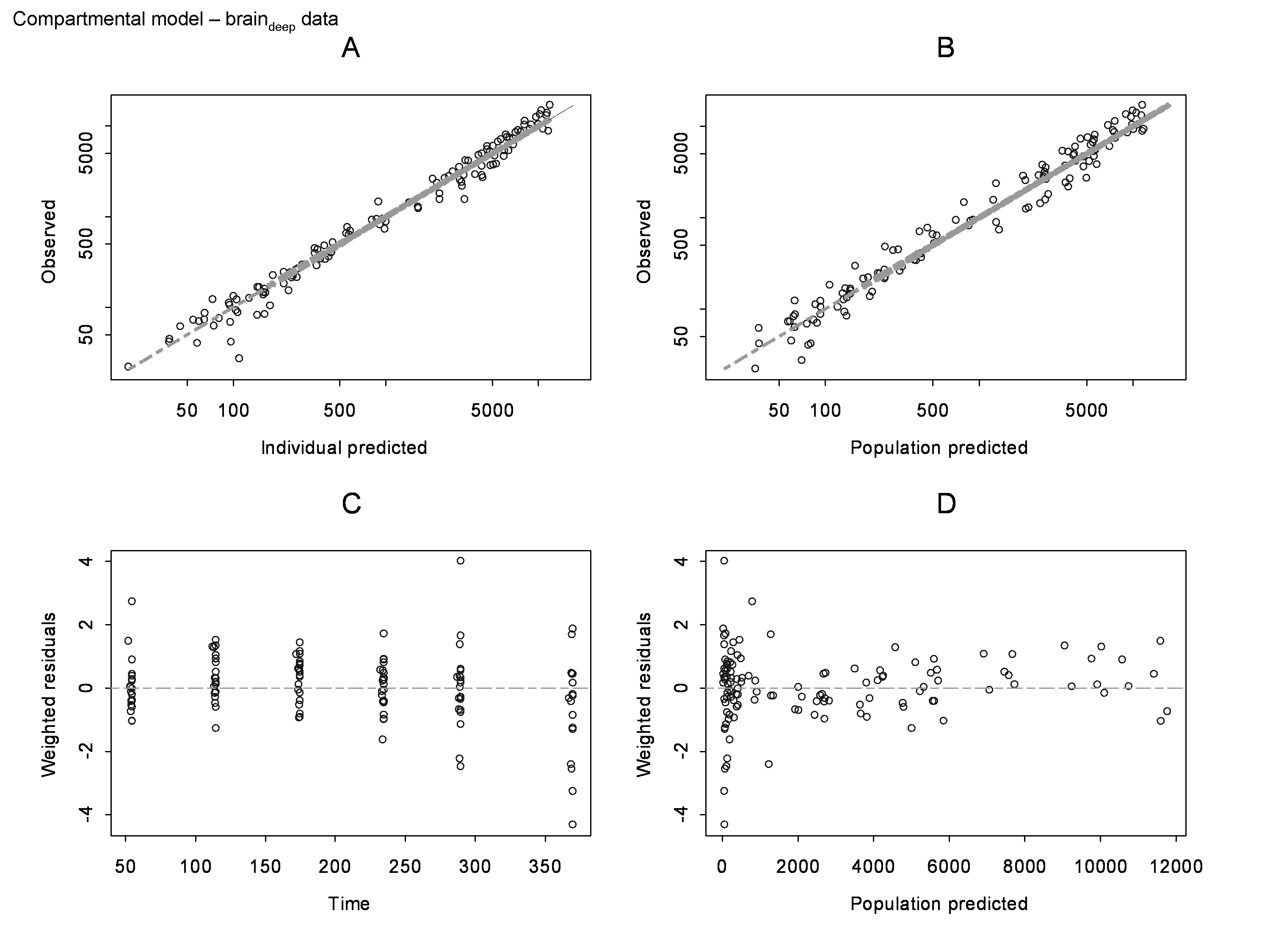

Supplement: Supplementary file 5 — Supplemental Fig. 5. The goodness of fit plot of the compartmental model for the braindeep data Supplementary material 5 (TIFF 582 kb) [file 10928_2013_9314_MOESM5_ESM.tif]

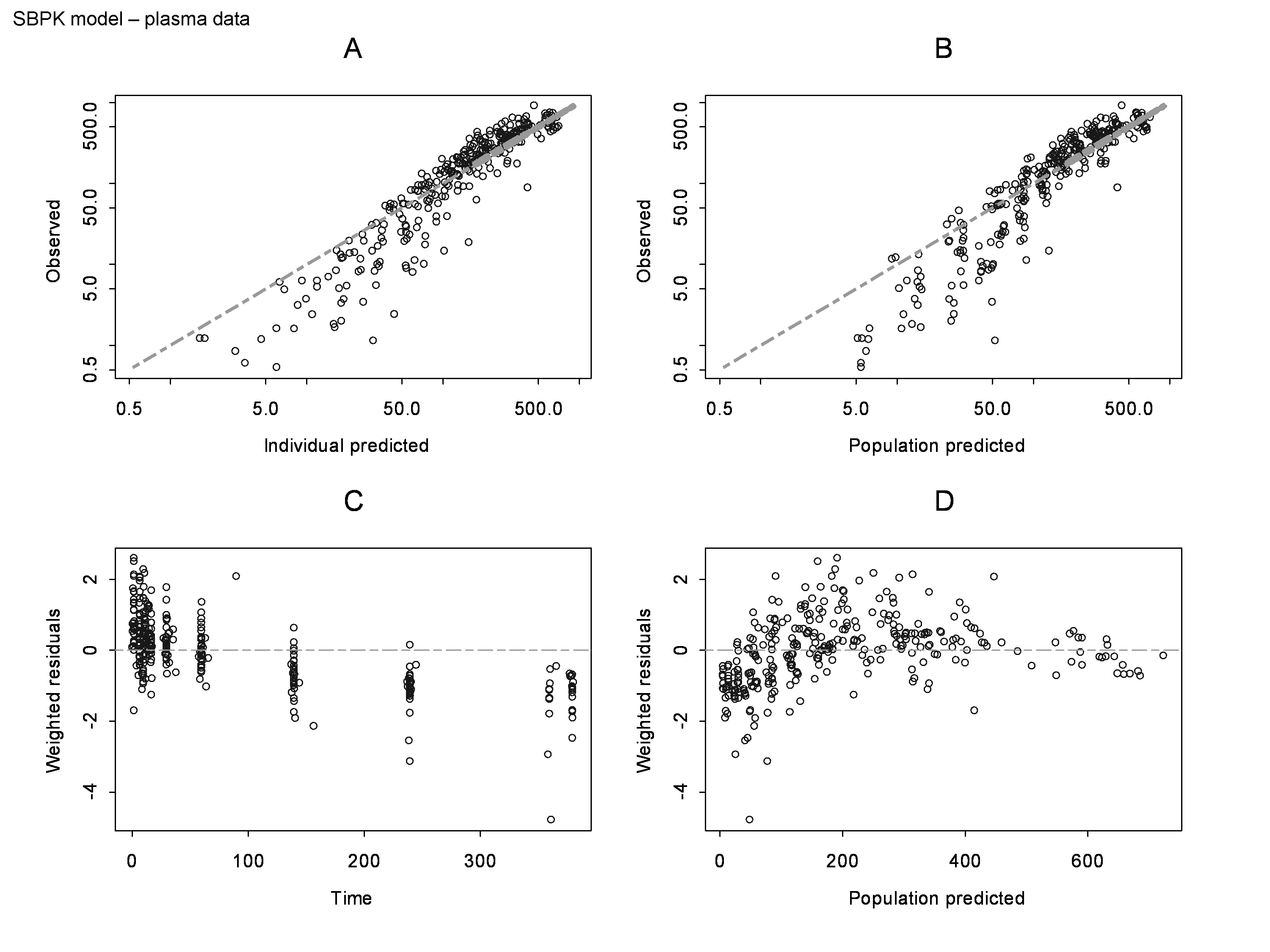

Supplement: Supplementary file 6 — Supplemental Fig. 6. The goodness of fit plot of the final SBPK model for the plasma data Supplementary material 6 (TIFF 602 kb) [file 10928_2013_9314_MOESM6_ESM.tif]

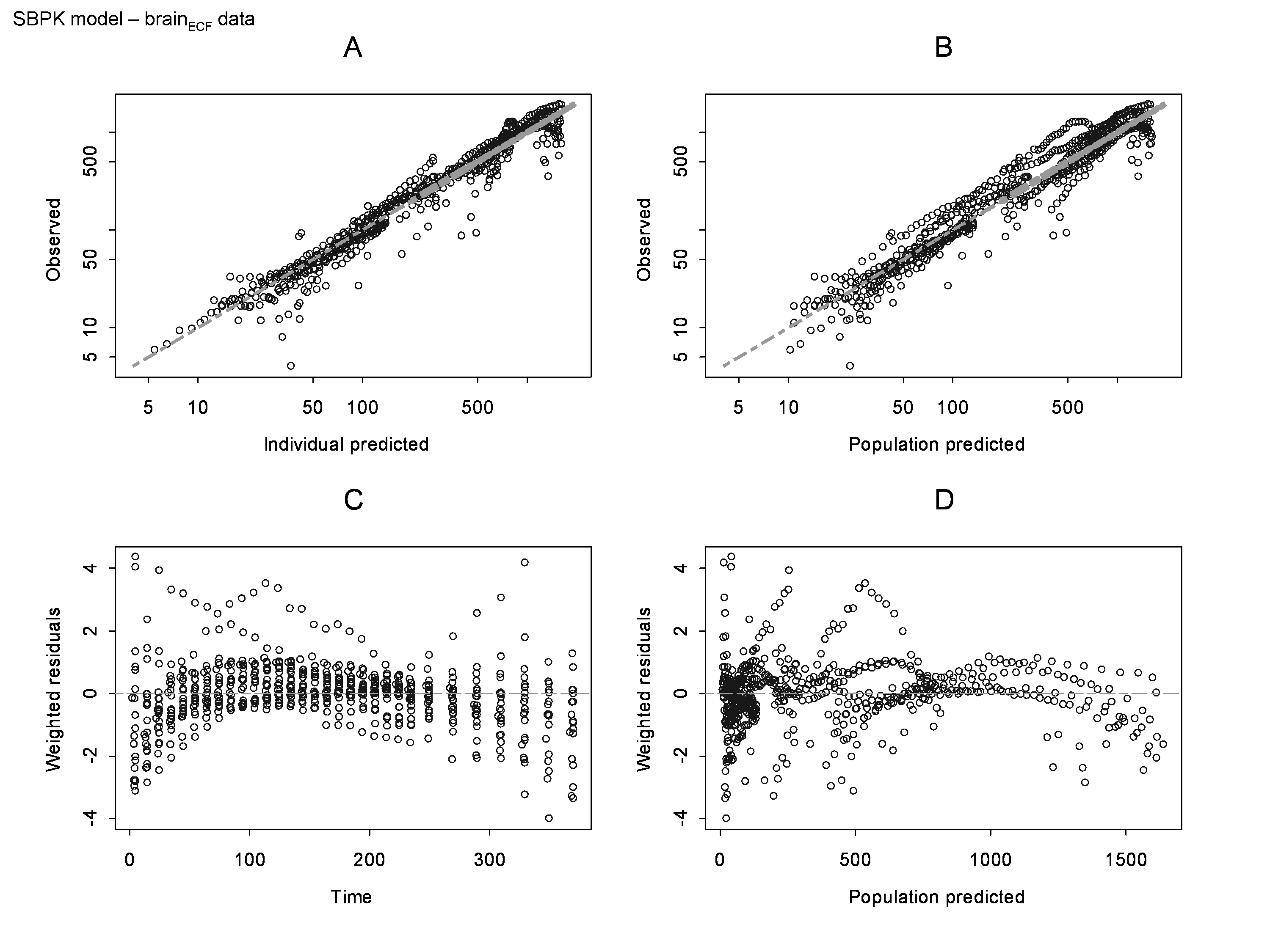

Supplement: Supplementary file 7 — Supplemental Fig. 7. The goodness of fit plot of the final SBPK model for the brainECF data Supplementary material 7 (TIFF 645 kb) [file 10928_2013_9314_MOESM7_ESM.tif]

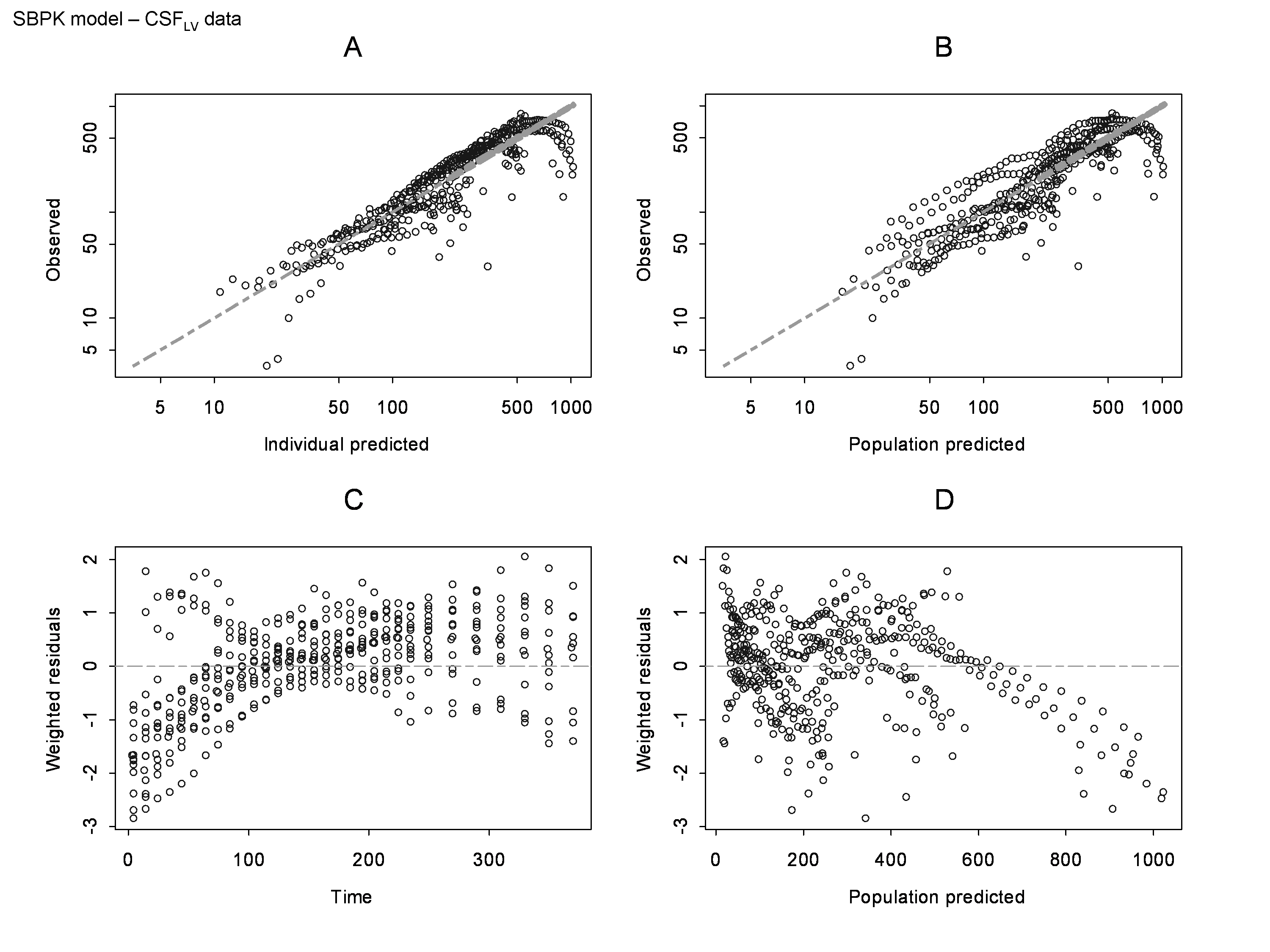

Supplement: Supplementary file 8 — Supplemental Fig. 8. The goodness of fit plot of the final SBPK model for the CSFLV data Supplementary material 8 (TIFF 638 kb) [file 10928_2013_9314_MOESM8_ESM.tif]

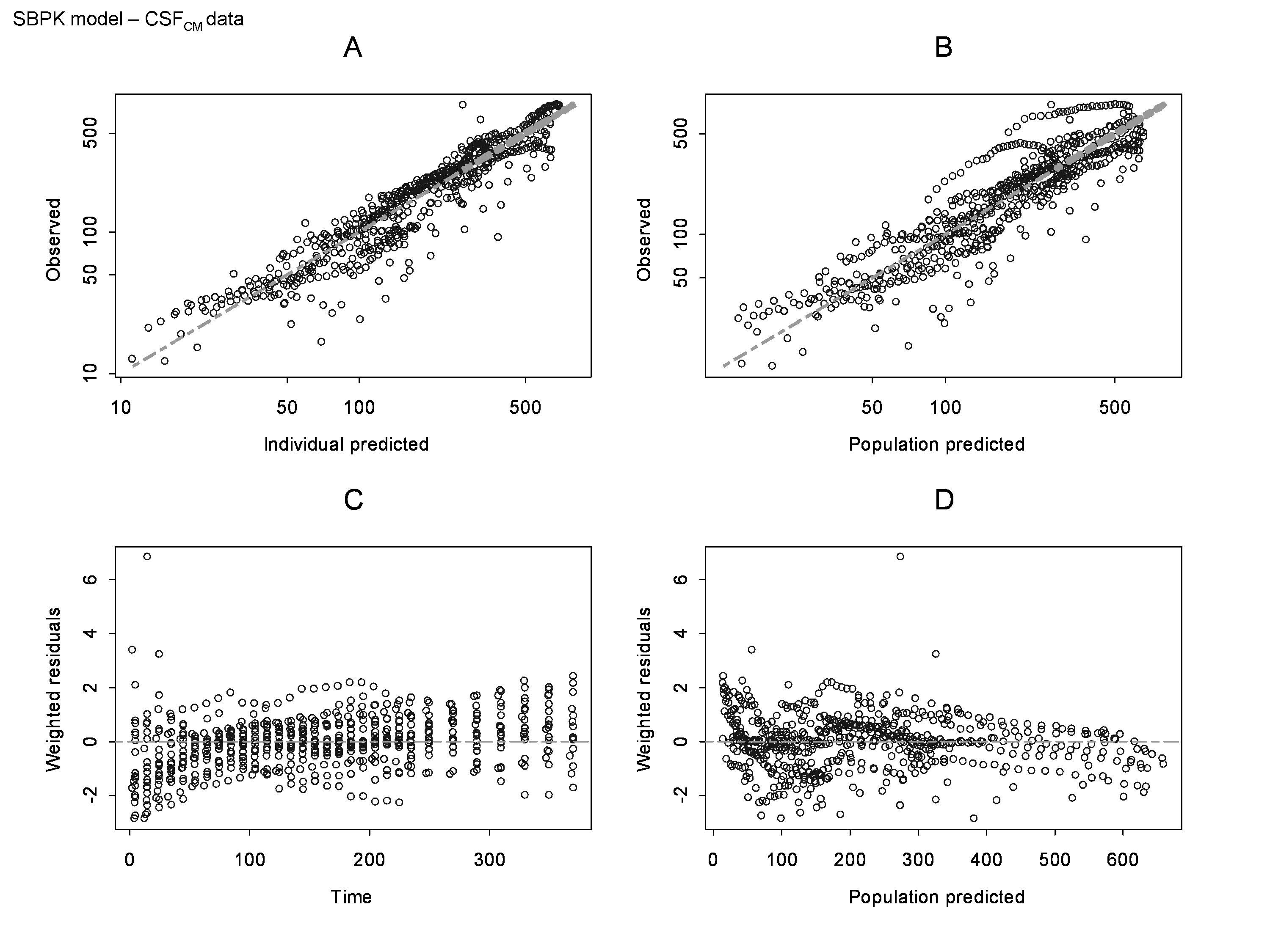

Supplement: Supplementary file 9 — Supplemental Fig. 9. The goodness of fit plot of the final SBPK model for the CSFCM data Supplementary material 9 (TIFF 657 kb) [file 10928_2013_9314_MOESM9_ESM.tif]

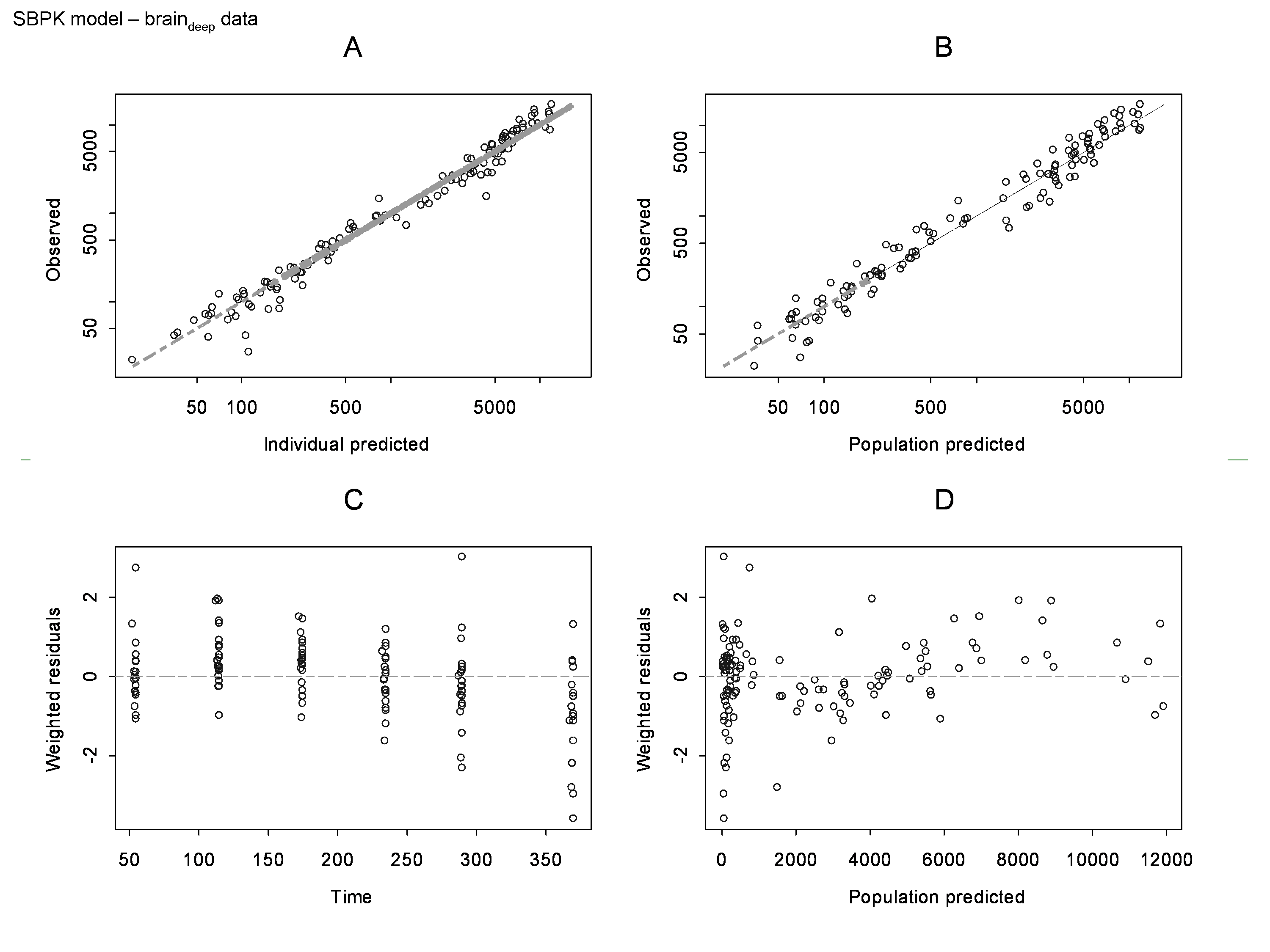

Supplement: Supplementary file 10 — Supplemental Fig. 10. The goodness of fit plot of the final SBPK model for the braindeep data Supplementary material 10 (TIFF 578 kb) [file 10928_2013_9314_MOESM10_ESM.tif]
